# Iceland-Faroe overflow revealed by in-situ observations

**DOI:** 10.1038/s41467-026-77220-w

**Published:** 2026-08-27

**Authors:** Karin Margretha H. Larsen, Guðrið Eriksdóttir, Bogi Hansen, Hjálmar Hátún, Steffen M. Olsen, Svein Østerhus

**Affiliations:** 1 Faroe Marine Research Institute, Tórshavn, Faroe Islands; 2 Danish Meteorological Institute, Copenhagen, Denmark; 3 NORCE Norwegian Research Centre and Bjerknes Centre for Climate Research, Bergen, Norway

**Keywords:** Physical oceanography, Physical oceanography

## Abstract

Overflow across the Iceland-Faroe Ridge is an important contributor to the deep limb of the Atlantic Meridional Overturning Circulation. Despite decades of observations, the strength, stability, and exact pathways of the overflow have remained uncertain. Here, we present results from several dedicated year-long current observations along the ~300 km long ridge crest. These observations reveal strong overflow at the central parts of the ridge with smaller contributions across both ends. The observations support the hypothesis that overflow is inhibited at both ends by upper-layer inflow of Atlantic water to the Norwegian Sea. In contrast, retroflection of Atlantic inflow over the central parts, allows the overflow to cross the ridge there, apparently as pulsating flow along two main pathways. The available data do not allow accurate estimates of volume transport, but they indicate that these pathways may well supply overflow in amounts consistent with previously published estimates.

## Abstract (web summary)

Observations along the Iceland-Faroe Ridge identify the central part of the ridge as the main pathway for overflow of cold, dense water feeding the deep limb of the Atlantic Meridional Overturning Circulation.

## Introduction

**Fig. 1:**
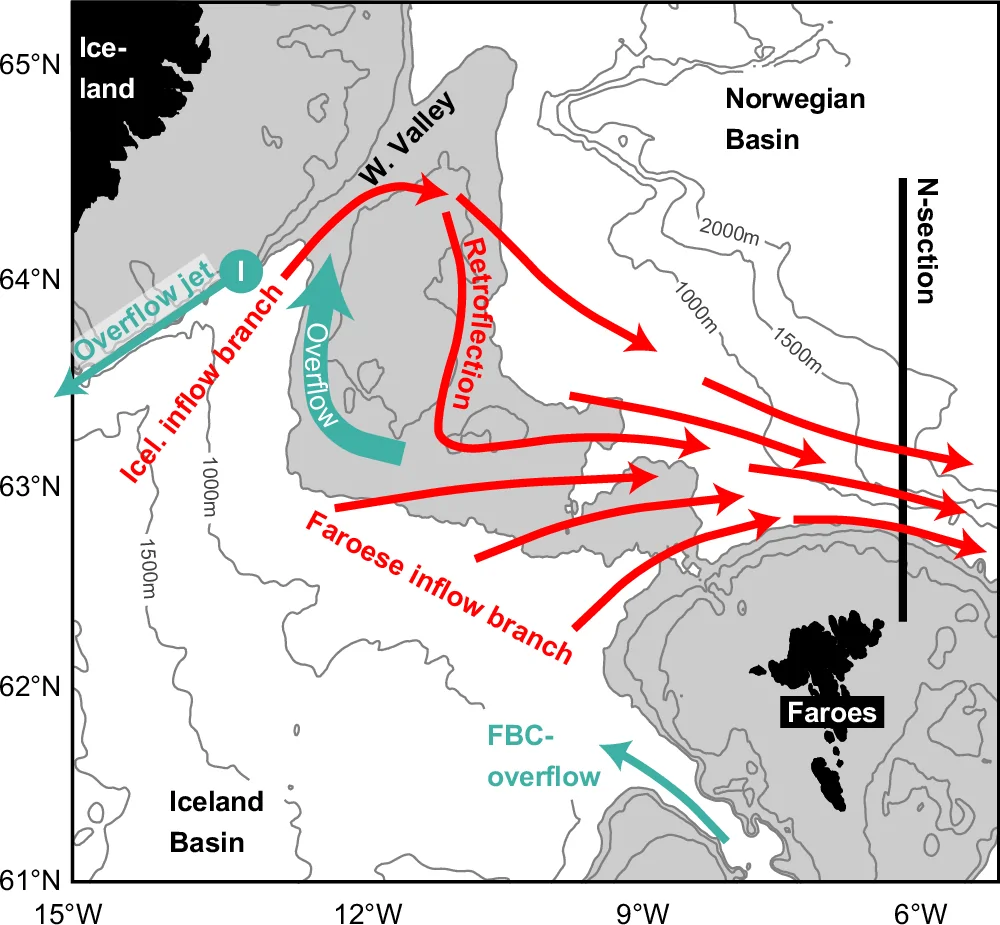
The Iceland-Faroe Ridge study region. Topographic map of the Iceland-Faroe Ridge region based on ETOPO1, where grey areas are shallower than 500 m and isobaths are at 200, 400, 500, 1000, 1500 and 2000 m. Red arrows show poleward pathways of warm Atlantic inflow across the Iceland-Faroe Ridge (IFR), mainly based on Hansen et al.^15^, showing the narrow Icelandic inflow branch (Icel. inflow branch) through the Western Valley (W. Valley), its retroflection back on the ridge and the broad Faroese inflow branch closer to Faroes. Turquoise arrows show cold equatorward overflow water, where FBC-overflow is the deep overflow from the Faroe Bank Channel. A turquoise circle with the letter I shows the location of a 2-year long ADCP (Acoustic Doppler Current Profiler) deployment over the south-Icelandic slope, which showed a strong bottom intensified jet in the direction of the turquoise arrow^7^. A curved turquoise arrow labelled Overflow indicates IFR-overflow water feeding into this jet^6,9^. A black line shows a hydrographic standard section north of Faroes, called the N-section.

The Iceland-Faroe Ridge (IFR) is part of a submarine ridge system extending from Greenland to Scotland, the Greenland-Scotland Ridge. This ridge system separates the deep areas of the Arctic Mediterranean (Arctic Ocean and Nordic Seas) and the North Atlantic. In the upper layers, around 8 Sv (1 Sv = 10^6^ m^3^ s^−1^) of warm and saline Atlantic water (Atlantic inflow, Fig. 1) flows into the Arctic Mediterranean, where it is cooled by the atmosphere. Its salinity is also reduced by precipitation, river runoff and by mixing with fresher water masses^1,2^, but the density still increases and ~70 % of the Atlantic inflow is transformed into a cold, dense water mass that fills up the deep parts of the Arctic Mediterranean^3^.

This deep reservoir of dense water results in a pressure difference at depth at the passages across the Greenland-Scotland Ridge driving overflow, balancing ongoing production of this water in the Arctic Mediterranean. This coupled system of warm Atlantic inflow in the upper layer and cold overflow at depth is a component of the Atlantic Meridional Overturning Circulation (AMOC), which is projected to weaken under future climate change^4^. Much effort has, therefore, been put into quantifying and monitoring the various components of this flow system.

In the literature, the overflow is described as composed of four branches with most of the water flowing through the Denmark Strait and the Faroe Bank Channel (FBC-overflow, Fig. 1) and a smaller fraction across the Wyville-Thomson Ridge. Mean transports and their variations have been fairly well described for these three branches^3^. In contrast, that is not the case for the fourth branch, the overflow across the Iceland-Faroe Ridge, IFR-overflow, which remains poorly constrained.

Research on the IFR-overflow has a long history, with early work reviewed by Hansen and Østerhus^1^. IFR-overflow was described already in the 19^th^ century, but received little attention until after 1950. Between 1959 and 1971, German research vessels occupied a hydrographic section 14 times along the southwestern flank of the IFR at bottom depths between 500 and 700 m, revealing that the flank was dominated by overflow water, although variably in time and space^5^. In 1960 and again in 1973, the International Council for the Exploration of the Sea (ICES) coordinated two international Overflow-experiments, where especially the Overflow-60 experiment focused on the IFR. Since then, several observational and modelling exercises have been conducted^6–8^, including a comprehensive study by repeated autonomous profiling glider transects over the southwestern flank^9^.

The early estimates of IFR-overflow volume transport, relying on hydrography and limited direct current observations on the ridge, estimated a total volume transport exceeding 1 Sv^6,10^. The more recent glider study^9^ found a lower bound of 0.8 Sv, although that includes Atlantic water entrained on route across the ridge. Nevertheless, temporal variations of transport and water mass characteristics still remain largely unknown.

The most perplexing unresolved question, however, is where the overflow water crosses the crest of the IFR. In the 2000s and 2010s, several observational campaigns, using Acoustic Doppler Current Profilers (ADCPs), were carried out along the ridge with the aim of identifying such pathways. Intuitively, the two ends of the ridge appear as plausible candidates from physical dynamical principles. The southeastern end, close to the Faroe slope, is the deepest part with bottom depths close to 500 m (Fig. 1). Observations at three ADCP sites in this region did indicate overflow, but it was weak and variable^11^.

At the northwestern end, near the Icelandic slope, the interface of the cold, deep water is typically found highest in the water column, making this location an obvious candidate. Support for this hypothesis is provided by the observation of a strong jet of overflow water over the Icelandic slope just downstream of the northwestern end of the IFR^6^. Based on glider observations, Beaird et al. concluded^9^, that the cold bottom water in the region of the overflow jet could only originate from the northwestern or central part of the IFR. The strength and stability of this jet was later confirmed by the results from an ADCP deployment (site I on Fig. 1) near the same location. The ADCP results confirmed the existence of a jet with a core 50 m above the bottom. Averaged over the more than 2-year long deployment, the core velocity of the jet was more than 50 cm s^−1^ along the bottom topography (turquoise arrow in Fig. 1) and throughout the entire record the weekly average core velocity remained above 30 cm s^−1^^7,12^. A cold, dense flow at these depths directly contributes to the lower limb of the AMOC^13^.

Directly upstream from the observed overflow jet is a narrow deep passage across the IFR, termed the Western Valley, with a sill depth around 425 m (Fig. 1). Direct current observations from the sill region in the early 1990s showed no evidence for significant flow through the Western Valley^6^. In an experiment from 2016 to 2017, an ADCP and two bottom temperature loggers were deployed for nine months at the sill. The results from this experiment largely confirmed the previous observations. Overflow water was observed to pass through the Western Valley, but the average transport was well below 0.1 Sv and the transport was variable^14^, ruling out that the overflow jet over the Icelandic slope is mainly fed by cold water continuously overflowing through the Western Valley.

The main reason for the low transport of overflow water through the Western Valley seemed to be the strong Atlantic inflow through the upper layers over the valley, which suppressed the overflow^14^. The same mechanism may also account for the weakness of the overflow over the southeastern end of the ridge^11^, since that region also has stable upper warm inflow^15^. However, over the central part of the ridge, there is a retroflection of the Atlantic inflow so that the Atlantic water in the upper layer flows along the overflow direction rather than against it (Fig. 1). This could explain why to expect overflow in this region. Previous studies have suggested that overflow branches along the IFR merge on the southwestern side of the ridge and that the combined overflow water is transported towards the region just downstream of the Western Valley^6,9^ (Fig. 1). Over the very steep slope region there, it is turned into a high-speed jet by gravity explaining the 50 cm s^−1^ flow strength observed there^7^. Without direct observational evidence, this remained hypothetical.

In this work, we present results from ADCPs with temperature loggers and dedicated hydrographic observations from the IFR to address the following questions: Are there locations on the IFR with sufficiently strong and stable overflow to explain the observed features of the overflow jet? Where are they, and what controls the overflow at these sites? We use observations from recent ADCP deployments at five sites on the ridge in the period May 2020 to June 2023 together with results from previously reported ADCP measurements on the ridge^11,14^. Combined, these data provide robust evidence for the configuration of overflow pathways across the ridge and a holistic overview of IFR-overflow.

## Results

### Hydrographic properties along the ridge crest

**Fig. 2:**
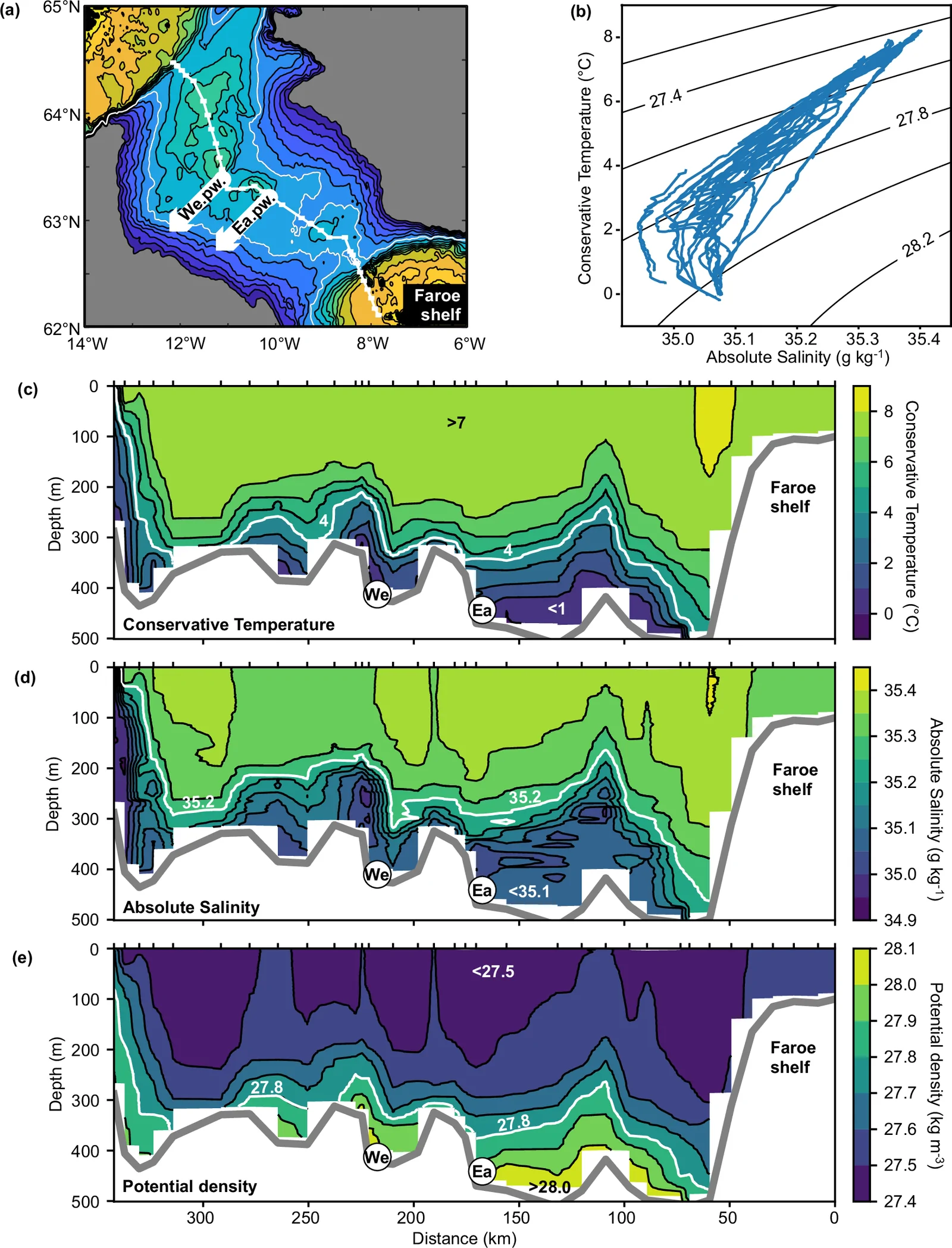
Hydrographic section along the Iceland-Faroe Ridge crest. **a** Topographic map of the Iceland-Faroe Ridge region based on ETOPO1. White rectangles connected by a white line show a section with 34 CTD (Conductivity, Temperature, Depth) stations occupied by R/V Jákup Sverri between the 21st and the 23rd of May 2021. **b** Blue traces show Conservative Temperature plotted against Absolute Salinity for each of the CTD profiles. Curved black lines show potential density anomaly in kg m^−3^. **c** Distribution of Conservative Temperature along the section with isotherms for every 1 °C. The 4 °C isotherm is enhanced in white. **d** Distribution of Absolute Salinity along the section with isohalines for every 0.05 g kg^−1^ and the 35.2 g kg^−1^ isohaline enhanced in white. **e** Distribution of potential density anomaly along the section with isopycnals for every 0.1 kg m^−3^ and the 27.8 kg m^−3^ isopycnal enhanced in white. Black ticks at the top of **c**–**e** indicate the locations of CTD stations. Locations of the two primary overflow pathways: the Western (We) and the Eastern (Ea) are shown in (**a**, **c**–**e**).

CTD (Conductivity, Temperature, Depth) observations were conducted during the ADCP deployment and recovery cruises. A section following the crest of the IFR (Fig. 2) shows the distribution of temperature, salinity, and potential density over the ridge during a cruise in May 2021. Both temperature and salinity exhibit short-term as well as long-term variations in this region, but Fig. 2 illustrates the typical situation over the IFR: In the upper layers, warm and saline waters dominate the section except for the region close to the Icelandic slope. Near the bottom, colder and less saline waters dominate, although the salinity distribution is rather patchy.

This patchiness is also evident in the TS-diagram in Fig. 2b. Straight traces indicate mixing between just two water masses: warm, saline Atlantic water in the upper layers and cold, fresher water at depth, which resembles the water around 500 m depth in the Norwegian Basin^1^. Other traces in Fig. 2b show intrusions between these two of water that is considerably fresher and derives from the region east of Iceland^2,9,16–18^. This results in a complicated and highly variable water mass distribution over the IFR, which will not be treated in any detail here.

In the classical literature^19^, overflow has often been defined as water with potential density exceeding 27.8 kg m^−3^. From Fig. 2e, most of the near-bottom water on the section, typically extending between 100 and 200 m up from the bottom, satisfies this criterion. Depending on the amount of low-salinity water in the water column, this density limit is reached at variable temperatures between 2 and 4 °C (Fig. 2b).

### Observations of overflow velocity

**Fig. 3:**
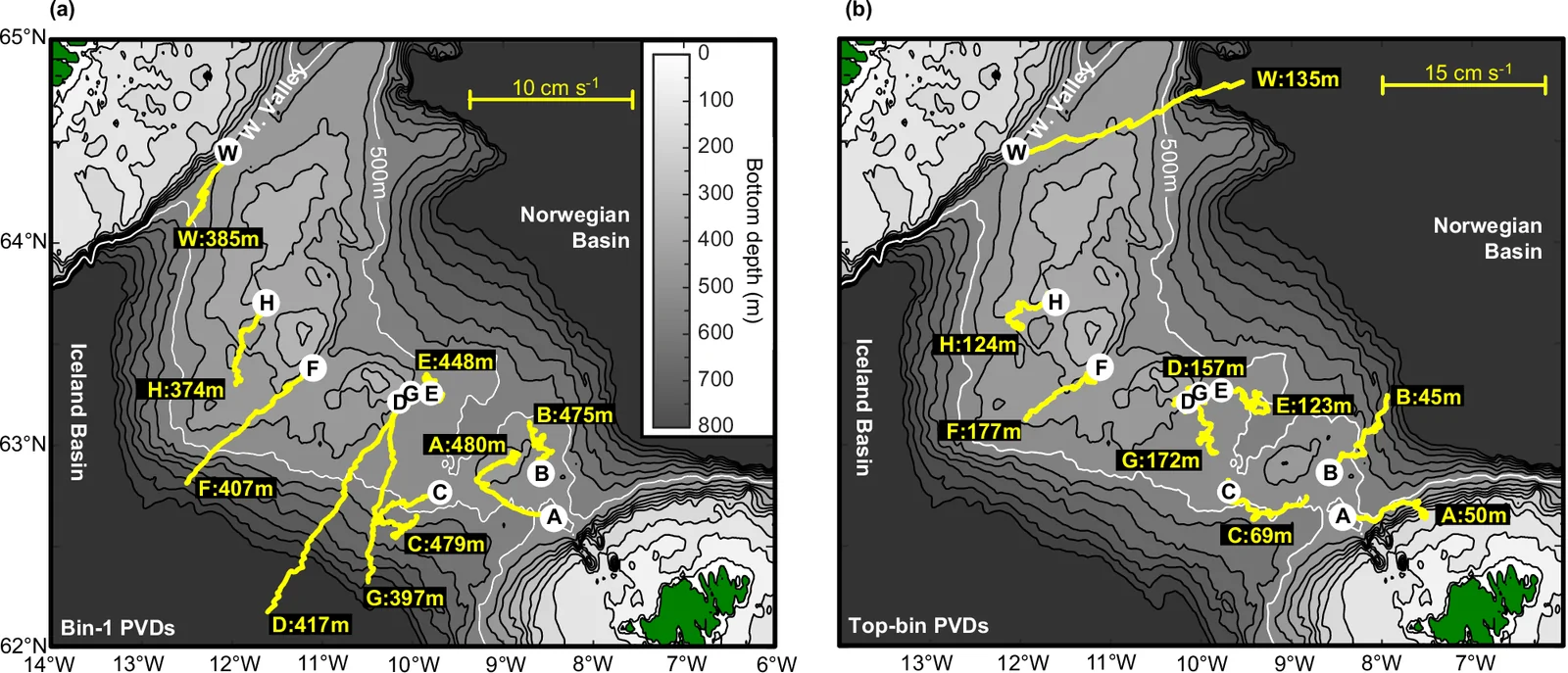
Progressive vector diagrams at deployment sites. **a** Progressive vector diagrams (PVDs) for the near-bottom overflows. **b** PVDs for upper layer Atlantic inflows. Background shading shows the bathymetry of the Iceland-Faroe Ridge based on ETOPO1 with isobaths for every 50 m down to 800 m depth. The 500 m isobaths are in white. White circles with letters show the nine Acoustic Doppler Current Profilers deployment sites. The yellow traces show PVDs based on daily averaged velocities for (**a**) the deepest bin, Bin-1, and (**b**) the Top-bin (see text and ‘Methods’). At the end of each PVD is shown the centre depth for (**a**) Bin-1 and (**b**) the Top-bin. The PVDs have been normalised by deployment duration so that they indicate the record-mean velocity as shown by the scales in both panels. Note that the scales in (**a**, **b**) are different in order to avoid too much overlap of the PVDs from different sites.

The main results of this study are from ADCP deployments at nine sites on the IFR. Each site is identified by a capital letter as shown on Fig. 3. Sites A, B, and C were occupied in 2003–2005^11^. Site W was occupied in 2016–2017^14^. The remaining sites were occupied in three periods: D and E in 2020–2021, F and G in 2021–2022, and H in 2022–2023. Here, we use daily averaged velocities measured by the upward looking ADCPs at several levels (bins) above the instrument. The deepest bin is denoted Bin-1. The shallowest bin with good data for every day of the deployment is denoted the Top-bin. See Supplementary Table 1 and ‘Methods’ for more details on deployments and processing of the data.

The yellow traces in Fig. 3a show progressive vector diagrams (PVDs) of the velocity from Bin-1, which can be considered an indicator for a potential overflow layer. Note that the PVDs do not represent Lagrangian paths for the overflow water, which will turn towards the northwest or north after crossing the ridge crest.

Indications of overflow are seen at all the sites except for B and E, but with variable strength and stability. The strongest signal is for site D where the Bin-1 velocity, centred 17 m above bottom, was 15 cm s^−1^ towards the Iceland Basin averaged over the whole deployment (362 days). The ADCP at site G was intended to represent the same overflow path, although it was moved 5.7 km from D, close to a sea-bed telecom cable to protect it from fisheries. Also, Bin-1 for G was centred farther (42 m) from the bottom. Taking these differences into account, the Bin-1 PVDs for the two sites are comparable. Hereafter, the overflow pathway at this location is termed the Eastern pathway. Site F likewise had very stable, but slightly weaker overflow and this pathway is hereafter termed the Western pathway. Indications of systematic overflow are also seen from the PVDs at sites H, and W, although weaker than at D and F. At sites A and C, the PVDs indicate overflow during the first part of the deployments but then, in January 2005, the flow at both of these sites turned towards the Norwegian Basin rather than towards the Iceland Basin.

The traces in Fig. 3b show PVDs for the Top-bin. Although they are not at the surface, they are quite consistent with the Atlantic water flow pattern in Fig. 1. At sites A, B, C, and W, they indicate inflow towards the Norwegian Basin, whereas the PVDs for F and H show fairly regular flow towards the Iceland Basin. The PVDs for the closely located sites D, E, and G in Fig. 3b show considerable variation (for D, the Top-bin PVD is so short that it is almost not visible). From Figs. 1 and 3, these sites are close to the southern boundary of the retroflection region of the inflow.

**Fig. 4:**
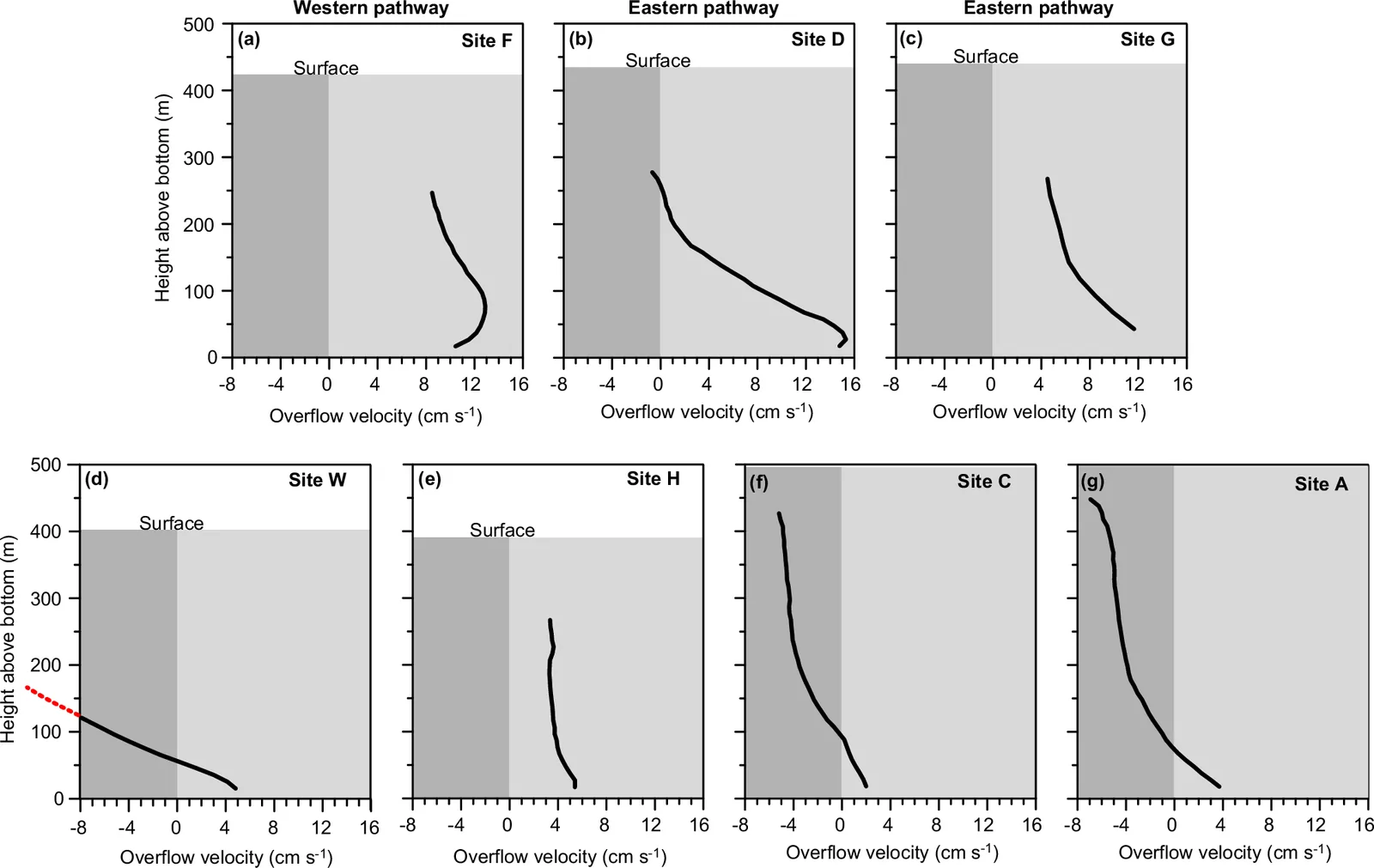
Velocity profiles at deployment sites. Average velocity profiles along the overflow direction (Table 1 and Supplementary Fig. 2) from Bin-1 to the Top-Bin, averaged over the individual deployment durations for (**a**) the Western pathway (site F), (**b**, **c**) the Eastern pathway (sites D and G, respectively), **d** site W, **e** site H, **f** site C and **g** site A (Supplementary Fig. 5 shows 14-day averaged velocity profiles for sites D, G, F and H). Velocities towards the Iceland Basin are illustrated with light grey background, whereas velocities towards the Norwegian Basin are on dark grey background. All the panels have the same vertical scale given as height above bottom at the individual sites. The horizontal (velocity) scale is also the same for all of the sites. The dashed red line for site W (**d**) indicates that the average velocity profile has been truncated to fit within the overflow velocity scale.

From the Bin-1 PVDs (Fig. 3a), seven of the ADCP sites (all but B and E) exhibit overflow, although with different regularity. For these overflow sites, we determine an overflow direction (see Methods). The velocity component along this direction is denoted the overflow velocity. We define the overflow stability as the percentage of days with positive Bin-1 overflow velocity. Main characteristics for the seven sites are listed in Table 1, where especially the average overflow velocity illustrates the dominance of the three main overflow sites: site F representing the western pathway and sites D and G that represent the eastern pathway. Profiles of the overflow velocity, averaged over the duration of each deployment, are shown in Fig. 4, where the three dominant sites are shown in the top panels.

**Table 1:** Characteristics of Acoustic Doppler Current Profiler observations at the seven overflow sites

ADCP Site	Bottom Depth	Deployment duration	Overflow direction	Average *U*_1_	Overflow stability	Transport per unit width up to		Overflow pathway
						100 m	200 m	
F	424 m	333 days	230°	10 cm s^−1^	76 %	12 m^2 ^s^−1^	24 m^2 ^s^−1^	Western
D	434 m	362 days	225°	15 cm s^−1^	87 %	13 m^2 ^s^−1^	20 m^2 ^s^−1^	Eastern
G	439 m	333 days	200°	12 cm s^−1^	84 %	10 m^2 ^s^−1^	19 m^2^ s^−1^	Eastern
W	402 m	278 days	220°	5 cm s^−1^	72 %	5 m^2 ^s^−1^	8 m^2 ^s^−1^	Minor
H	391 m	369 days	205°	5 cm s^−1^	74 %	6 m^2 ^s^−1^	12 m^2^ s^−1^	Minor
C	497 m	324 days	230°	2 cm s^−1^	56 %	6 m^2 ^s^−1^	10 m^2^ s^−1^	Minor
A	498 m	259 days	295°	4 cm s^−1^	65 %	5 m^2 ^s^−1^	8 m^2 ^s^−1^	Minor

Average *U*_1_ is the Bin-1 overflow velocity averaged over the deployment period. Transport per unit width up to either 100 m or 200 m above bottom is defined in Methods.

### Observations of bottom temperature at ADCP sites

**Fig. 5:**
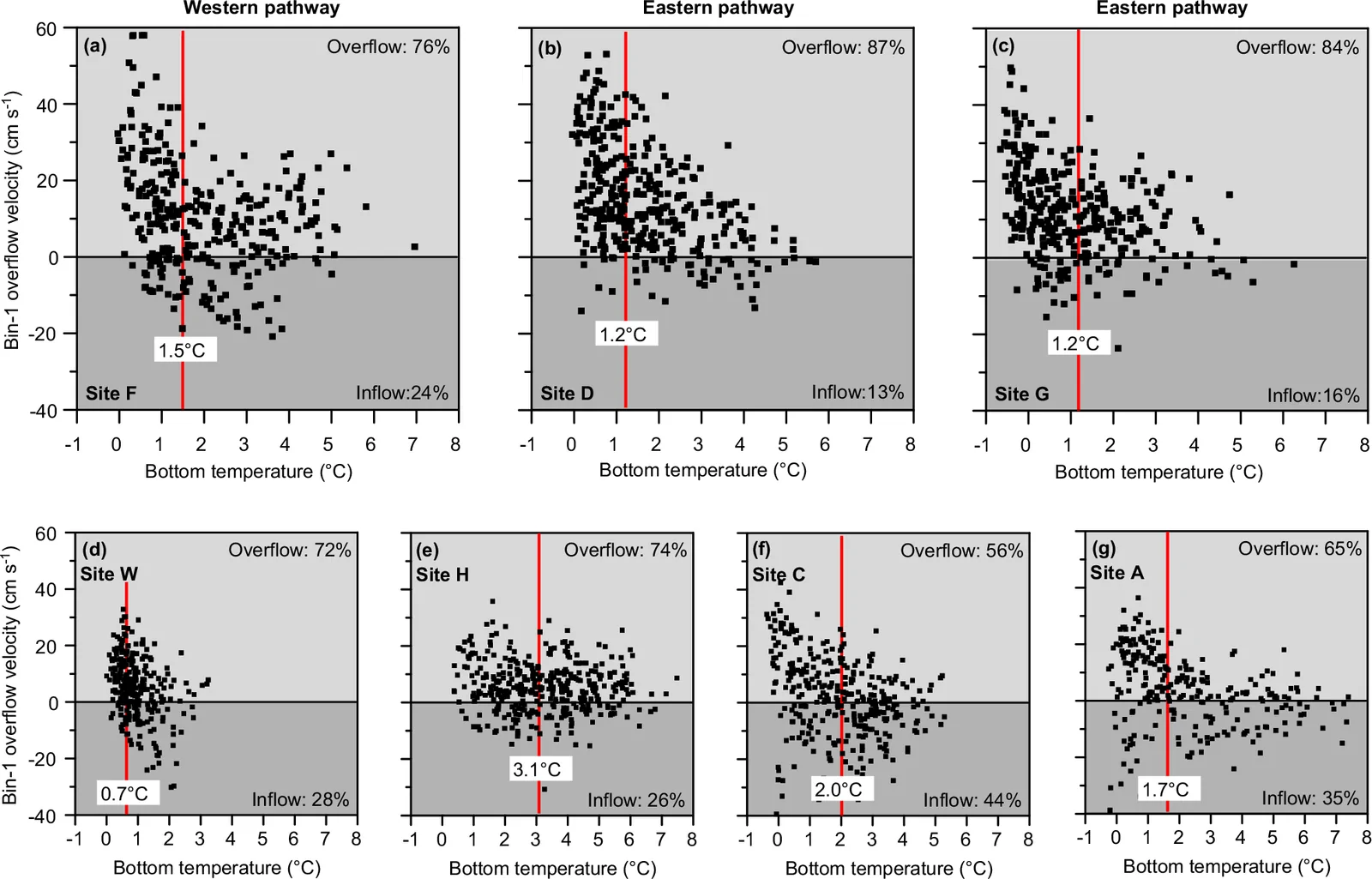
Bottom temperature and overflow velocity at deployment sites. Black squares show daily averaged Bin-1 overflow velocity plotted against daily averaged bottom temperature for (**a**) the Western pathway (site F), **b**, **c** the Eastern pathway (sites D and G, respectively), **d** site W, **e** site H, **f** site C and **g** site A. On each panel is shown the percentage of days with positive overflow velocities (overflow stability – light grey background) and negative overflow velocities (inflow – dark grey background). Vertical red lines show the median bottom temperature. **a**–**c** Show the three dominant overflow sites representing the two main overflow pathways.

At each of the nine deployment sites (Fig. 3), temperature was logged at least every 20 min throughout the deployment period. The temperature loggers were located at most 6 m from the bottom and we will refer to the measurements as bottom temperature. In Fig. 5, daily averages of bottom temperature and the Bin-1 overflow velocity are compared for the overflow sites with special emphasis on the three dominant sites representing the two main pathways. Although the figure shows considerable scatter, the highest overflow velocities are only seen for low bottom temperatures at all sites, except for site H. At least half of the time, the bottom temperature for most of the deployments in Fig. 5 was at or below 2 °C, indicating overflow (Fig. 2b).

### Pulsating overflow

**Fig. 6:**
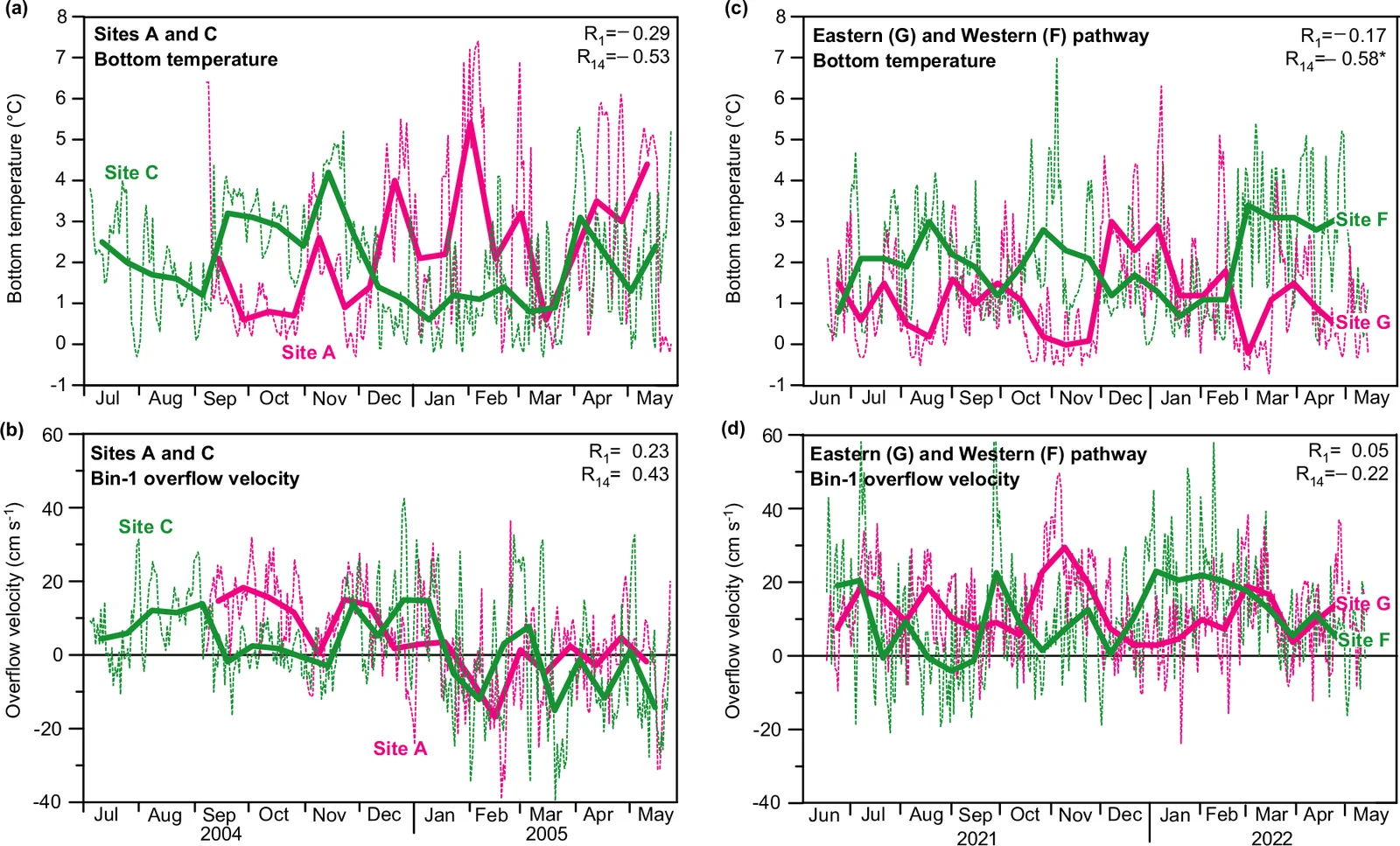
Time series of bottom temperatures and overflow velocities. **a** Bottom temperature and **b** overflow velocity at sites A (magenta) and C (green) throughout their deployment periods. **c** Bottom temperature and **d** overflow velocity at the Eastern (magenta) and Western (green) pathways throughout their deployment periods. Thin dotted lines show daily averages and thick continuous lines show 14-day averages. Numbers in upper right corners show correlation coefficients for daily averaged data (R_1_) and for 14-day averages (R_14_). A * for R_14_ in (**c**) indicates statistical significance with 95 % confidence with serial correlation taken into account (see ‘Methods’). Sample sizes were 259 days for (**a**, **b**) and 333 days for (**c**, **d**).

In order to look at spatial and temporal variability, we analyse data from two ADCP pairs with overlapping periods. At sites A and C, the two ADCPs were overlapping during the period from 7 September 2004 to 23 May 2005. Similarly, the ADCPs at the Western pathway (site F) and Eastern pathway (site G) were deployed on the same day and were also recovered on the same day the following year. In Fig. 6, bottom temperature and Bin-1 overflow velocity for each of these two pairs of concurrent observations are compared.

At all of these four sites, daily averaged values for bottom temperature as well as overflow velocity (thin dotted lines in Fig. 6) vary considerably, which shows that the overflow occurs in pulses at all of the sites. The 14-day averaged values (thick continuous lines in Fig. 6) hint at some relationships. For bottom temperature (Fig. 6a, c), there appears to be an inverse relationship between the two ADCPs in each pair and correlation coefficients are negative. For the overflow velocities, there seems to be a direct relationship between A and C (Fig. 6b), whereas the two main pathways (F and G) seem to vary inversely (Fig. 6d). This could indicate that the overflow over the central part of the ridge has a tendency to switch between the Western and the Eastern pathway. These links are vague, however, and only for the 14-day averaged bottom temperatures at the Western and Eastern pathways is the correlation coefficient (*R* = –0.58*) significantly different from zero with 95 % confidence.

### Observations of overflow downstream of the ridge crest

**Fig. 7:**
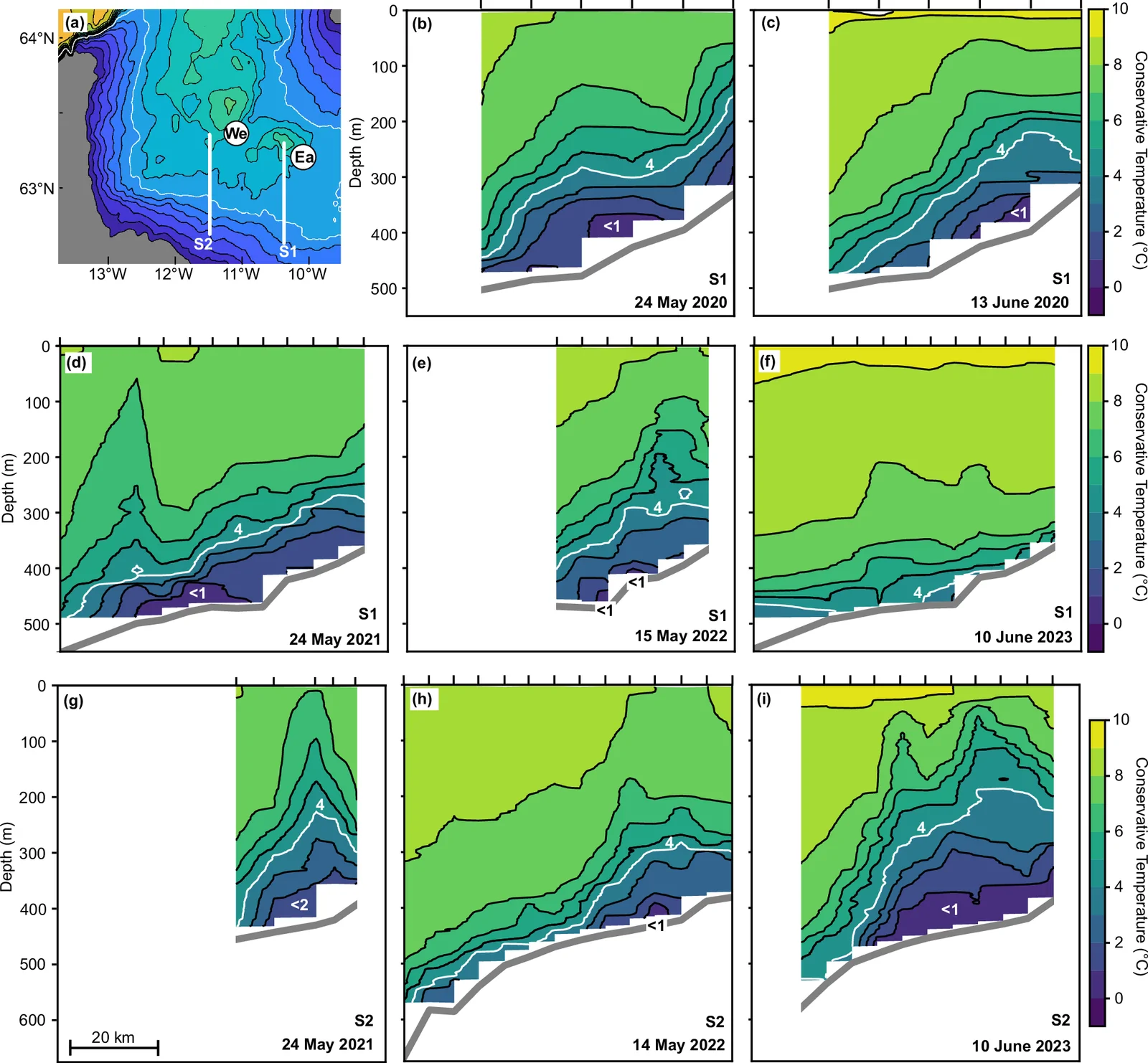
Hydrographic sections on the southwestern flank of the Iceland-Faroe Ridge. Conservative Temperature on repeated occupations of two hydrographic sections, S1 and S2, shown on the map in (**a**) together with the location of sites at the Eastern (Ea) and Western (We) pathways. **b**–**f** Show results from five occupations of S1. **g**–**i** Show three occupations of S2. All the section plots have the same scale and colour coding for Conservative Temperature. Isotherms are shown for every 1 °C and the 4 °C isotherm is enhanced in white. All the section plots have the same vertical (depth) and horizontal (shown in (**g**)) scales. Absolute Salinity for these sections is shown in Supplementary Fig. 6.

To investigate the further fate of the overflow water crossing the ridge, repeated CTD-observations were conducted along two meridional sections, S1 and S2, shown on the map in Fig. 7a. Section S1 was occupied five times (Fig. 7b–f) and all but one of these show water colder than 1 °C over the southwestern flank of the IFR. On section S2 (Fig. 7g–i), two of the three occupations show water colder than 1 °C while the third has water colder than 2 °C. The cold cores have temperatures consistent with the bottom temperatures observed at the overflow sites (Fig. 5).

As a whole, these sections demonstrate the typical occurrence of water, sufficiently cold to be classified as overflow water downstream of the ridge crest. Although some of the uppermost parts may return to the Norwegian Sea, parts of this water will continue as an overflow current southwest of the ridge crest. From the variability of the CTD sections, this is not a steady current, but rather a pulsating flow consistent with Fig. 6. As for Fig. 2, the sections in Fig. 7 indicate the typical height of the overflow layer to vary between 100 and 200 m up from the bottom.

### Overflow volume transport

Ideally, the ADCP data could be used to estimate volume transport of the overflow past each of the sites. This would involve vertically integrating the overflow velocity from ADCP profiles and multiplying by an appropriate width. The vertical integral should be from the bottom up to the top of the overflow layer as defined by e.g. the 27.8 kg m^−3^ isopycnal or an isotherm. For the deployment at site W, it was possible to make rough transport estimates but there, the experiment included bottom temperature loggers on both sides of the ADCP, approximately covering the width of the overflow, including one higher up on the Icelandic slope^14^. This allowed integration of overflow velocity up to a given (estimated) isotherm, and also allowed a rough estimate of the appropriate width. For the other overflow sites, we do not have that information, which makes realistic estimates more difficult.

For all of the overflow sites, we have calculated time series of the overflow transport per unit width obtained by vertical integration of the overflow velocity from the bottom up to specified levels. To focus on overflow, only positive overflow velocity values were included in the integration (see Methods). This would be appropriate if all the water passing the ADCP in the overflow direction ended up as overflow. In reality, some of the overflow water passing the ADCP will return towards the Norwegian Sea. This tends to make the integrals overestimates, but probably not so much for the main overflow sites (D, G and F), where the Bin-1 overflow velocity indicates only few (and weak) reversals (Fig. 5).

The upper limit for these vertical integrals should be the top of the overflow layer, as determined from hydrography. From the CTD data (Figs. 2 and 7), this level is often seen between 100 and 200 m above the bottom, but most likely, it is highly variable, as indicated by the high variability of the bottom temperature (Fig. 6). For the three dominant overflow sites (D, G and F), high values of the overflow transport per unit length are biased towards low bottom temperature (Supplementary Fig. 1). This implies that a few events with high overflow layers may contribute disproportionately to the average transport, which makes the choice of an upper limit even more difficult.

With the available information, we cannot determine an appropriate upper limit for the vertical integration on any given day during any of the deployments (except for site W) or how much of the water below this level is sufficiently dense or cold to be characterised as overflow. We can, however, get some impression on the order of magnitude by choosing different values for the upper limit. For that purpose, Table 1 lists record-mean averages of the overflow transport per unit length using two different choices for the upper limit: 100 m above bottom and 200 m above bottom, indicated by the CTD sections (Figs. 2 and 7). Here, the estimates in Table 1 for sites D and G, representing the same pathway, are quite similar for the two choices, supporting the choices and the assumption on the pathway.

To convert these estimates to volume transport, an appropriate overflow width is needed. From basic principles within rotational hydrodynamics, this width is not expected to be much less than the internal (baroclinic) radius of deformation, which over the IFR is on the order of 10 km^20^. The two overflow pathways were located over relatively steep bottom inclines with the ADCP sites D, G and F located close to the deep ends of the inclines (Fig. 2). If the main overflow currents are linked to these inclines, the overflow width for these sites should not be much more than 20 km. For the Eastern pathway, this is supported by the lack of overflow at site E (Supplementary Fig. 2), 12 km from site G and 18 km from site D.

Combining these two alternatives for the overflow width with the values in Table 1, the average overflow transport in the Eastern pathway is found to range between 0.1 Sv (10 km × 10 m^2^ s^−1^) and 0.4 Sv (20 km × 20 m^2^ s^−1^). For the Western pathway, a similar range is indicated: 0.1 Sv to 0.5 Sv. The large ranges mean that we are not able to determine average volume transport with any reasonable level of accuracy. The apparent anti-correlation between the two pathways (Fig. 6) likely makes the summed transport of the two more stable than for either of them individually.

### Controlling mechanisms

Having established the two dominant pathways for IFR-overflow, the question arises, what the mechanisms are that control the overflows along them. As documented in the previous section, a major drawback is the lack of sufficiently frequent information on the hydrographic structure above the ADCP sites. This makes the variations of the height of the overflow layer and its transport difficult to determine with any reasonable degree of accuracy.

Acknowledging these limitations, we have not tried to explore all aspects of the time series acquired or their interrelationships, but have confined ourselves to a set of simple correlation analyses reported in Table 2. The correlation coefficients in this table are between four parameters represented by time series from each of the three dominant overflow sites. The two overflow indicators are the bottom temperature, *T*_B_, and the Bin-1 overflow velocity, *U*_1_ (Fig. 5). As might be expected, these two parameters are negatively correlated at all of the sites: positive overflow velocity is linked to low bottom temperature. Although statistically significant, the correlation coefficients are not numerically high, for which panels (a–c) in Fig. 5 provide an explanation: high values for *U*_1_ only occur when *T*_B_ is low, but *U*_1_ may also be low or even negative when *T*_B_ is low.

**Table 2:** Correlation coefficients between daily averaged overflow indicators and overflow predictors

Parameters	W. pathway	Eastern pathway	
	Site F	Site D	Site G
	333 days	362 days	333 days
*T*_B_ versus *U*_1_	−0.29**	−0.48***	−0.36**
*T*_B_ versus *h*	+0.65***	+0.55***	+0.45**
*T*_B_ versus *U*_T_	n.s.	+0.33**	n.s.
*U*_1_ versus *h*	−0.43**	−0.36**	−0.39*
*U*_1_ versus *U*_T_	−0.51***	−0.63***	−0.45***

The first column shows the two parameters being correlated. *T*_B_ is the bottom temperature. *U*_1_ is the Bin-1 overflow velocity. *U*_T_ is the eastward-component of the velocity of the Top-bin. *h* is the corrected SLA-value at the altimetry grid point closest to the ADCP position (see Methods). Sample size is listed as the number of days below each site id. Significance levels are indicated by asterisks: One asterisk, *, means that the correlation coefficient is different from zero with 95 % confidence (*p* < 0.05), where serial correlation has been taken into account (see Methods). ** means significance with 99 % confidence (*p* < 0.01). *** means significance with 99.9 % confidence (*p* < 0.001). Correlation coefficients that were not significant with 95 % confidence have been replaced by n.s.

The other two parameters in Table 2 are overflow predictors, which are likely to control or be linked to the overflow. One of these is the strength of the Atlantic inflow in the upper layer above the ADCP site, which has been suggested to reduce or block the overflow^14^. After some experimentation, the eastward velocity component of the Top-bin, denoted *U*_T_, was chosen to represent the Atlantic inflow strength for all of the ADCP sites.

Another likely predictor for overflow is the height of the interface between Atlantic and overflow waters over the site. Direct observations of this height are only sparse, but observations from the neighbouring N-section (Fig. 1) have previously shown^15^ highly significant positive correlations between sea level anomaly and the depth of the interface between warm Atlantic and cold overflow water likely caused by geostrophic adjustment. We therefore use the Sea Level Anomaly (SLA) at the altimetry grid point closest to the ADCP position for this parameter, labelled *h* in Table 2. The high short-term variability observed for the overflow (e.g. Fig. 6) indicates that we should focus on mesoscale sea level variations. The values for *h* have therefore been corrected by subtracting the spatial average over a large area at each time step (see ‘Methods’). As a further justification for this approach, the magnitudes of most of the correlation coefficients in Table 2 increased substantially by the correction.

The last four rows of Table 2 confirm that both of the two chosen overflow predictors, *h* and *U*_T_, are linked to the overflow at each of the dominant overflow sites. Highly significant positive correlations between *T*_B_ and *h* are interpreted to mean that a low (mesoscale) sea level over the ADCP site is associated with a high interface, as suggested above, and that a high interface is associated with a low bottom temperature, as might be expected. The negative correlations between *U*_1_ and *h* then imply that a high interface (low sea level) also is associated with high Bin-1 overflow velocity. Lagged correlation analyses do not indicate any clear phase lags between *h* and either *T*_B_ or *U*_1_. Without clear lags, the causality of these relationships is less evident, but Table 2 clearly demonstrates that a high interface is associated with strong overflow, involving both low temperature and high overflow velocity close to the bottom.

The other overflow predictor, *U*_T_, may be weakly related to the bottom temperature, *T*_B_, at site D, but not for the other sites. It is, however, clearly related to the Bin-1 overflow velocity *U*_1_. When the Atlantic inflow is strong over one of the ADCP sites, the overflow velocity close to the bottom is likely to be weak. Again, there is no indication of any clear lags in these relationships.

## Discussion

The dataset presented here constitutes a comprehensive spatial coverage of ADCP observations over the crest of the IFR with several long-term mooring sites along the whole ridge. In planning the ADCP sites, we aimed at covering all the likely overflow passages, but we cannot guarantee this. Despite that caveat, the observations provide a consistent picture. We confirm, as noted in the introduction, that the overflow is weak and variable at both the southeastern (sites A, B, and C) and the northwestern (site W) end of the ridge. Instead, we find the strongest and most stable overflow over the central part of the ridge. Our results support the conjecture^14^ that the lack of strong overflow at both ends of the IFR is linked to the strong Atlantic inflows in these regions, which explains why the main overflow pathways are located in the retroflection region of the Atlantic layer flow (Fig. 1).

From our observations, the main overflow pathways across the ridge are the Eastern (sites D and G) and Western (site F) pathways (Fig. 3a). At sites D and G, we observed the strongest average Bin-1 overflow velocity (Table 1) and positive overflow velocity extended high above the bottom on average (Fig. 4). Also, these two sites had the highest overflow stability and the lowest median bottom temperatures of all the overflow sites except for site W (Fig. 5). At site F, we also observed a relatively strong average Bin-1 overflow velocity (Table 1), which increased in strength up to 75 m above the bottom (Fig. 4). The median bottom temperature was, however, higher than at the Eastern pathway and the stability lower (Fig. 5 and Table 1).

Overflow is also likely to occur at site H, but this site is shallower, has higher bottom temperatures (Fig. 5e), and weaker overflow velocities (Table 1). Summarising, our observations indicate two main overflow pathways, i.e. the Eastern and Western pathways. In addition, overflow crosses the IFR over its southeastern end (sites A and C), through the Western Valley (site W), and over site H, but these three minor overflow pathways are weaker and less stable.

Having water with overflow characteristics passing over a monitoring site on the IFR does not necessarily imply that it will continue as overflow contributing to the AMOC. However, the temperature sections (Fig. 7) document that cold overflow water is typically present over the southwestern flank of the ridge, at least during summer. From there, it is not likely/dynamically plausible for overflow water to return to the Norwegian Sea and this water may be the main source for the overflow jet observed over the Icelandic slope (Fig. 1), consistent with the suggestion by Perkins et al.^6^. On its way across the ridge, the overflow water is mixed with the Atlantic water above. As the water progresses, the overflow layer will contain increasing amounts of entrained Atlantic water^13^.

From glider observations of the typical along-isobath speed^9^, overflow water over the southwestern flank of the IFR will require 1-2 weeks to propagate from sections S1 and S2 (Fig. 7) to the exit of the Western Valley from where the steep bottom slope is expected to funnel and accelerate the flow^9^. This residence time over the southwestern IFR flank acts as a buffer, smoothing the short-term pulses seen at the ADCP sites (Fig. 6 and Supplementary Fig. 3), which may help explain the stable character of the overflow jet over the Icelandic slope. Nevertheless, the effect of the overflow pulses remains, and the flow over the southwestern IFR flank should not be seen as a continuous homogenous current (Supplementary Fig. 4).

With the lack of sufficiently frequent hydrographic data from the water above the ADCP sites, we have not been able to determine typical values for overflow volume transport for the dominant sites or pathways better than within a factor of five. We can conclude, however, that the two main pathways, together with smaller contributions from the other locations^9,14^, may well combine to a total IFR-overflow transport approaching 1 Sv, especially if entrainment downstream of the ridge crest is included. This estimate is comparable to previous observation-based estimates^6,9,10^.

The highly significant correlations listed in Table 2 document that the overflows along the two main pathways are linked to both the strength of the Atlantic inflow in the water above (negatively) and to the height of the interface between Atlantic and overflow water (positively). How these links may be expressed more quantitatively in terms of overflow volume transport remains an open question as do the causal aspects of these relationships. We suggest that the most fruitful strategy for future research needs to involve high-resolution numerical modelling of the IFR. The observations presented here will be essential for validating model simulations. The simulations themselves are, however, beyond the scope of this paper.

Although the IFR-overflow is weaker than the overflows through the Denmark Strait and the Faroe Bank Channel^3^, it is still considered to be an important contributor to the deep limb of the AMOC^13^. Monitoring the transport and characteristics of this overflow branch is therefore an integral part of monitoring the AMOC, but also a difficult one. With present-day technology, monitoring on the ridge would require several traditional moorings with temperature and preferably salinity loggers at several depths. However, they are unlikely to survive the heavy fishing activity for long. An alternative approach would be to monitor the overflow jet over the Icelandic slope, although distinguishing between IFR-overflow and FBC-overflow may require additional research.

## Methods

### Acoustic Doppler Current Profiler (ADCP) data

The ADCP data are from deployments at nine sites on the IFR. At each site, an ADCP has been deployed with durations between 259 and 369 days (deployment details are in Supplementary Table 1). The upward looking ADCPs were either placed directly on the bottom in trawl-protected frames or moored in buoys anchored 6 m above the bottom (Supplementary Table 1). All ADCPs sampled at 20 min intervals, with one ping per ensemble for the BroadBand ADCPs and 12 pings per ensemble for the LongRanger ADCPs. The velocity data from each ensemble are organised into vertical bins, which for these deployments had bin lengths of either 10 m or 25 m (Supplementary Table 1). With increasing distance from the instrument, the velocity data become progressively contaminated by errors. After recovery, the data are quality controlled and invalid ensembles from each bin are error-flagged. To de-tide the velocities, a harmonic tidal analysis is run for each bin on the validated velocities from the whole deployment using an adapted version of the Foreman FORTRAN package^21^. From the harmonic constants, the tidal current is predicted for each ensemble and subtracted from the measured values. Daily-averaged velocities are then calculated for each bin by averaging the de-tided non-flagged velocities.

The ADCPs include temperature sensors that logged the ambient temperature every 20 minutes. Additional high-quality temperature loggers from Sea-Bird (SBE 37 or SBE 56) were attached to all the ADCPs except for at sites A and B. The high-quality temperature loggers, which logged the temperature at least every 20 min, have uncertainties less than 0.01 °C. The internal ADCP temperature sensors are less accurate, but intercalibration with high-quality sensors verifies that their uncertainties are less than 0.1 °C, which is sufficient for our purpose. These temperatures are reported as in-situ temperatures.

### Determination of overflow parameters

To check whether the velocity data from the ADCPs exhibited some preferred directions towards the Iceland Basin, Rose diagrams were generated for the lowest bins. For each bin, these diagrams show the fraction of days with current direction within 5° classes. If the diagrams for the first four bins showed a preferred direction consistent with overflow, this determined the overflow direction. Supplementary Fig. 2 shows one example of an ADCP site with a clear overflow direction (site D) and one site (site E), for which no clear overflow direction could be selected. The sites with clear overflow directions are termed overflow sites (Table 1). For these sites, the overflow velocity of a given bin on a given day is defined as the velocity vector projected onto the overflow direction. Two estimates of transport per unit width are determined by integrating interpolated overflow velocity from the bottom up to 100 m and 200 m above the bottom, respectively, but only including values with positive overflow velocity.

### CTD data

CTD data were acquired by a Sea-Bird 911 + CTD with carousel water sampler. Water samples were routinely acquired on every profile and analysed on land with a salinometer for calibrating salinity. Post cruise, the CTD data were quality controlled, averaged to metre interval, and calibrated according to the TEOS-10 standard (www.TEOS-10.org).

### Satellite altimetry data

The altimetry data were selected from the global gridded (0.25°×0.25°) sea level anomaly (SLA) field available from Copernicus Marine Environment Monitoring Service (CMEMS) (http://marine.copernicus.eu): SEALEVEL_GLO_PHY_L4_MY_008_047. For each of the ADCP sites, daily SLA-values were extracted from the closest grid point. The distance between the ADCP position and the closest grid point varied between 0 and 14 km. As argued in the text, the SLA values were corrected by subtracting at every time step (day) the spatial average of SLA at all grid points between 58.125°N and 65.875°N in latitude and between 19.875°W and 4.875°E in longitude.

### Statistical methods

Correlations between two data sets are estimated by the Pearson correlation coefficient. To account for serial correlation in the data, the statistical significance of correlation coefficients is corrected by the modified Chelton method^22^. Significance is indicated by asterisks: * means *p* < 0.05. ** means *p* < 0.01. *** means *p* < 0.001. No asterisk means *p* > 0.05.

## Supplementary information

Supplementary Information

Transparent Peer Review file

## Data Availability

The in-situ datasets utilised in the current study are available from https://envofar.fo/index.php?page=climate.

## Code availability

In this work we have used MATLAB, Python, Grapher and Fortran code to analyse and plot the data displayed in this paper. This code is available from the corresponding author upon request.
