# Supplementary material for: Iceland-Faroe overflow revealed by in-situ observations
Source: Nat Commun. 2026 Aug 27;17:10257. doi: 10.1038/s41467-026-77220-w (PMC13616910; doi:10.1038/s41467-026-77220-w)
Supplement: Supplementary file 1 [file 41467_2026_77220_MOESM1_ESM.pdf]

Iceland-Faroe overflow revealed by in-situ observations

Karin Margretha H. Larsen[1], Guðrið Eriksdóttir[1], Bogi Hansen[1], Hjálmar Hátún[1], Steffen M. Olsen[2], Svein Østerhus[3]*

[1]Faroe Marine Research Institute, Nóatún 1, Tórshavn, Faroe Islands.

[2]Danish Meteorological Institute, Sankt Kjelds Plads 11, Copenhagen, Denmark

[3]NORCE Norwegian Research Centre and Bjerknes Centre for Climate Research, Jahnebakken 5, Bergen, Norway.

Supplementary Table and Figures

In this supporting information we provide one additional table and six additional figures that support the results in the main text.

Supplementary Table 1. Details of the ADCP deployments. "LR": LongRanger ADCP in trawlproof frame. "BB150": BroadBand 150 kHz ADCP in trawlproof frame. "BB 75": BroadBand 75 kHz ADCP in buoy. The trawlproof frames were deployed directly on the bottom. The "BB 75" ADCPs were moored 6 m above the bottom. All the ADCPs were upward looking and sampled at 20 minute intervals.

	ADCP	Position		Period	Dur.	Bin	Depth to		
Site	Model	Latit.	Longit.	yyyymmdd-yyyymmdd	Days	length	Bott.	Bin-1	Top-bin
A	LR	62.6350	-8.4533	20040907-20050523	259	10m	498m	480m	50m
B	LR	62.8611	-8.5893	20030705-20040610	342	10m	495m	475m	45m
C	LR	62.7634	-9.7116	20040704-20050523	324	10m	497m	479m	69m
D	BB150	63.2159	-10.1420	20200525-20210521	362	10m	434m	417m	157m
E	BB75	63.2463	-9.7977	20200613-20210521	343	25m	490m	448m	123m
F	BB150	63.3749	-11.1229	20210616-20220514	333	10m	424m	407m	177m
G	BB75	63.2407	-10.0434	20210616-20220514	333	25m	439m	397m	172m
H	BB150	63.6989	-11.6165	20220605-20230608	369	10m	391m	374m	124m
W	BB150	64.4450	-12.0627	20160815-20170519	278	10m	402m	385m	135m

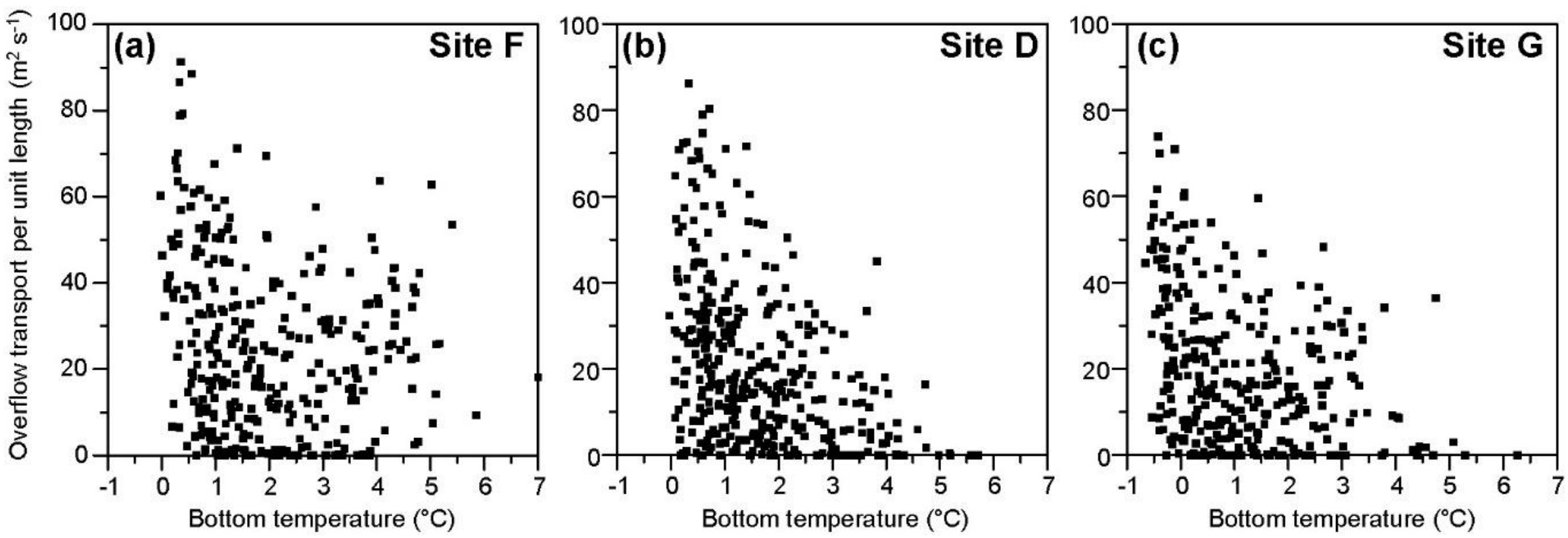

Supplementary Fig. 1. Bottom temperature and overflow transport. Black squares show daily averaged overflow transport per unit length up to 200 m above the bottom plotted against daily averaged bottom temperature for the three main overflow sites: (a) Site F, (b) Site D and (c) Site G.

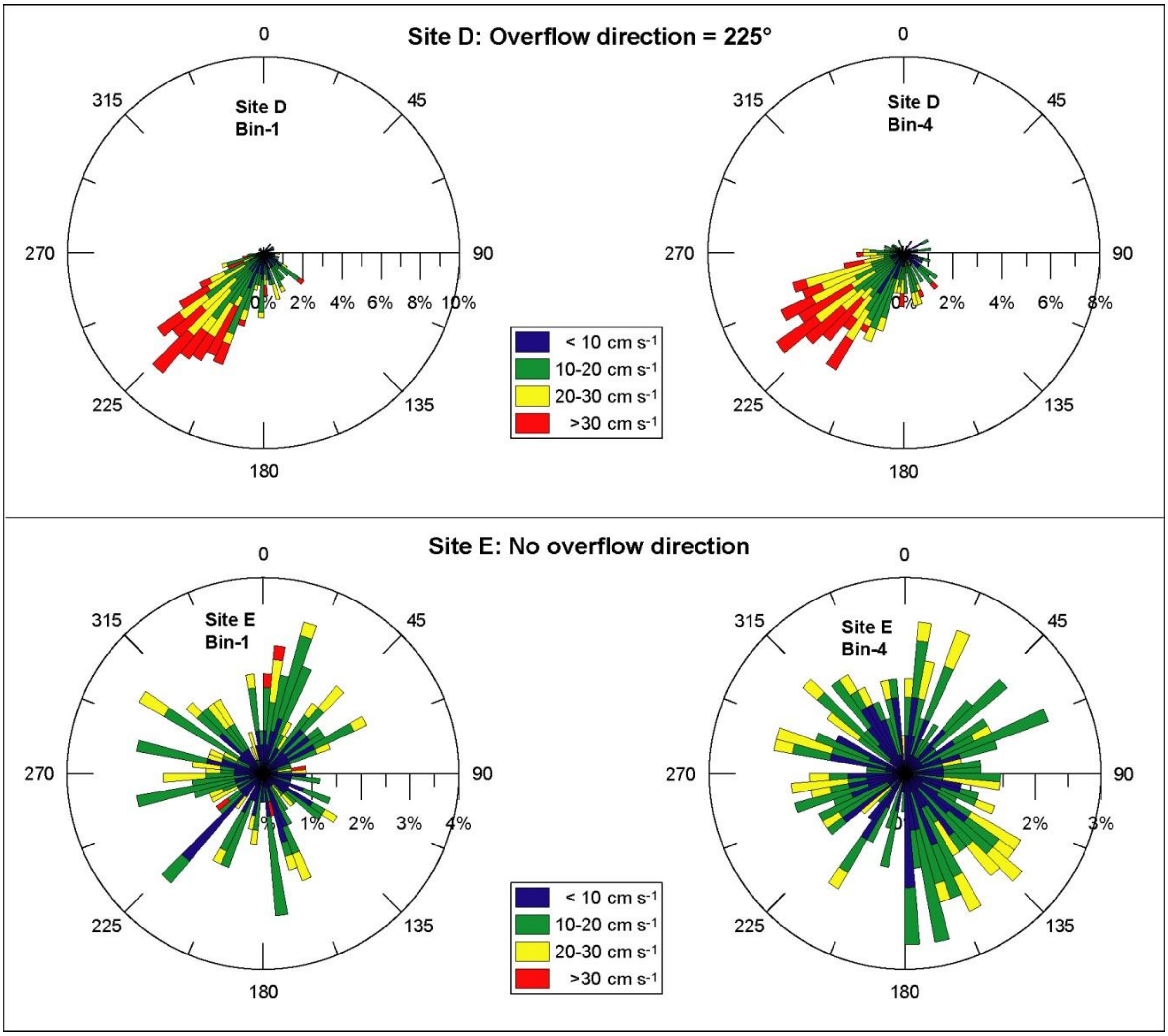

Supplementary Fig. 2. Rose-diagrams of velocity direction from two deployment sites. Examples of Rose-diagrams used to determine overflow direction at each site, if applicable. For each diagram, the length of each spoke shows the fraction of days, for which the velocity of that bin was within the 5° directional range indicated. The colours indicate the speed distribution within the directional range. The top two diagrams illustrate site D, for which a well-defined overflow direction may be determined. The bottom diagrams illustrate site E where no specific overflow direction seems to be preferred.

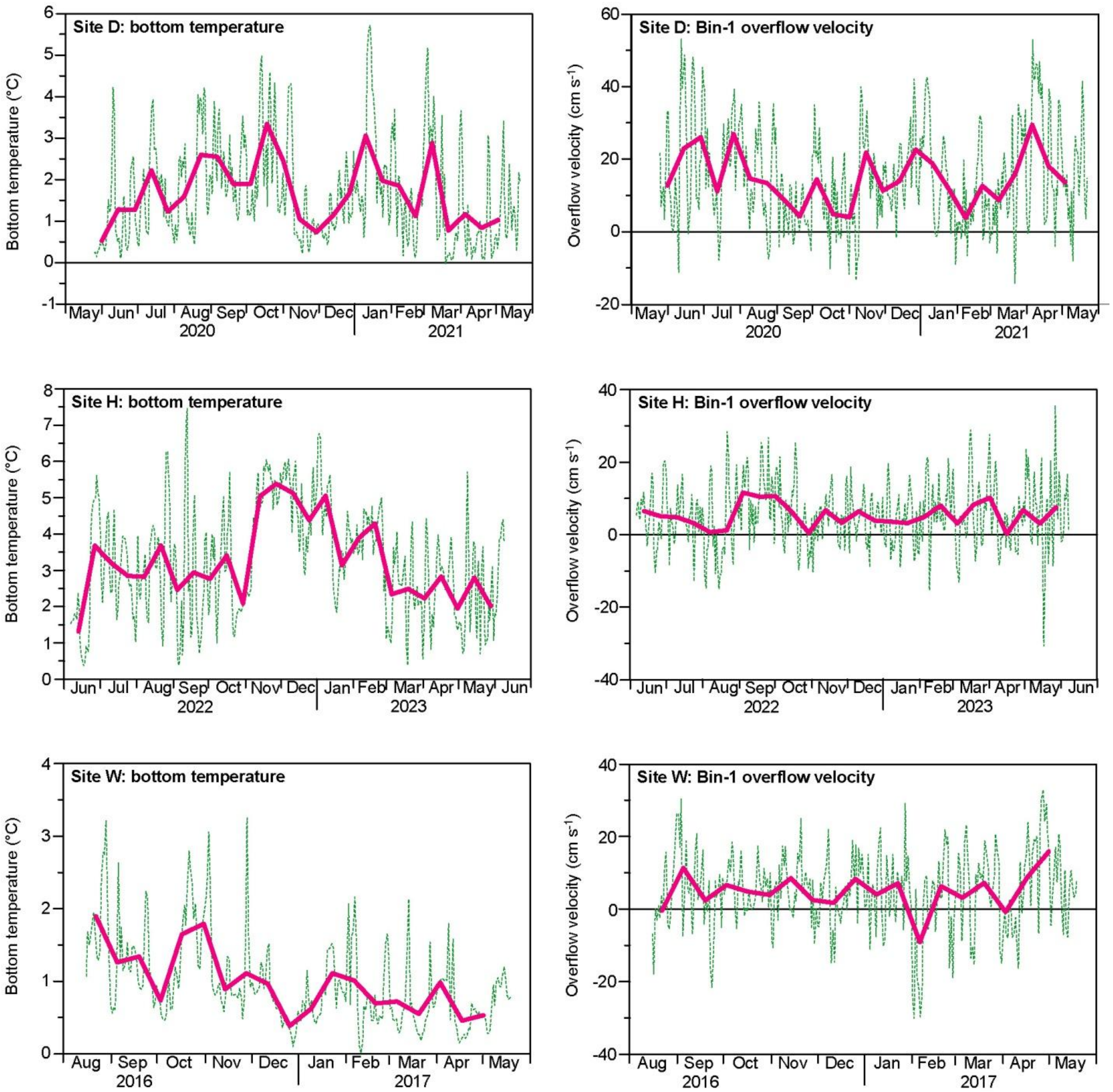

Supplementary Fig. 3. Time series of bottom temperatures and overflow velocities. Bottom temperature and Bin-1 overflow velocity at sites D, H, and W throughout their deployment periods. Thin dotted green lines show daily averages. Thick continuous magenta lines show 14-day averages.

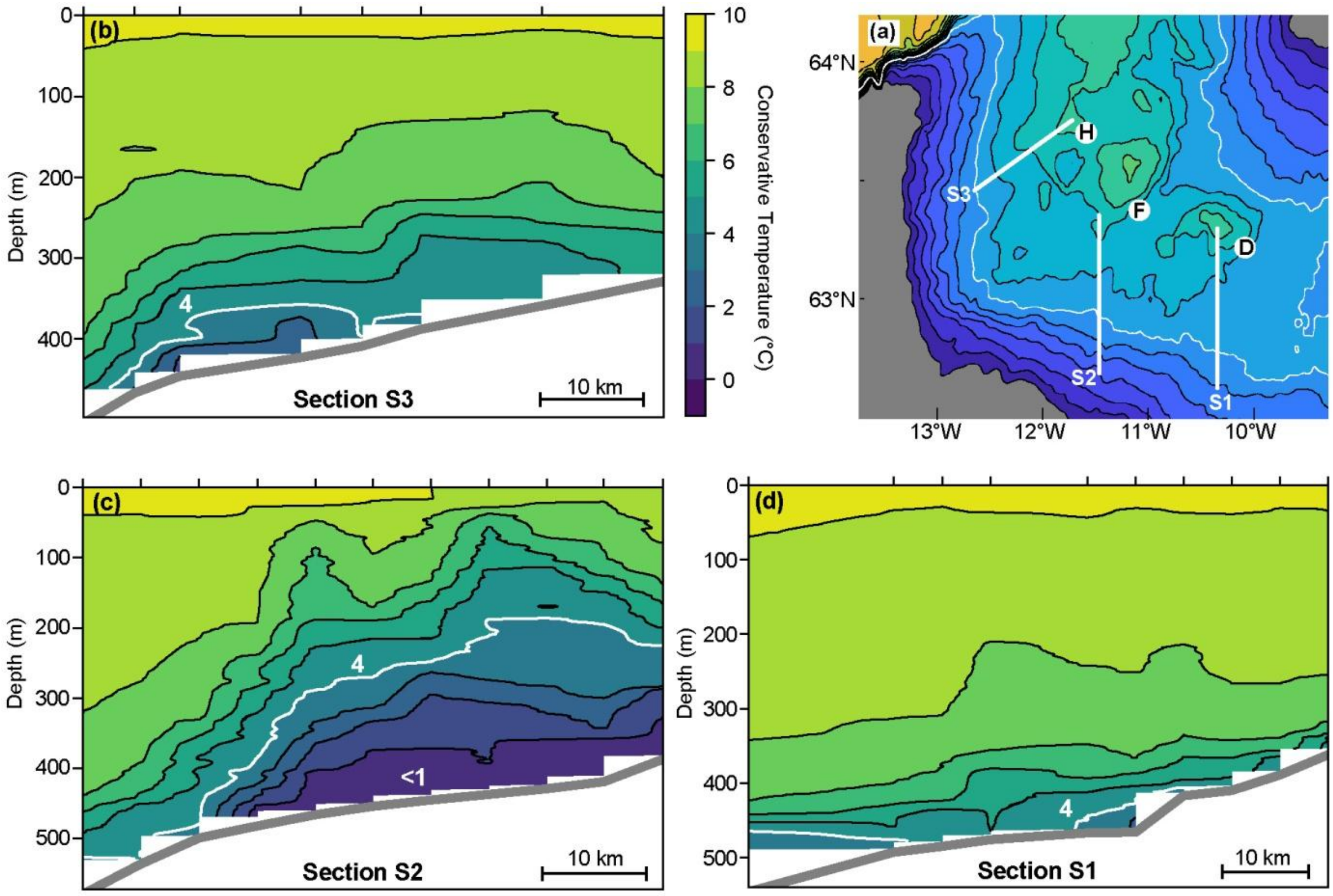

Supplementary Fig. 4. Three hydrographic sections on the southwestern flank of the Iceland-Faroe Ridge. (a) Map showing the location of three hydrographic sections occupied on cruise 2328 by R/V Jákup Sverri from 9 June to 11 June 2023. Panels (b-d) show Conservative Temperature at sections S3, S2 and S1, respectively. The three section plots have the same vertical (depth) and horizontal (shown in panel (c)) scales and have the same scale and colour coding for Conservative Temperature. Isotherms are shown for every 1 °C and the 4 °C isotherm is enhanced in white.

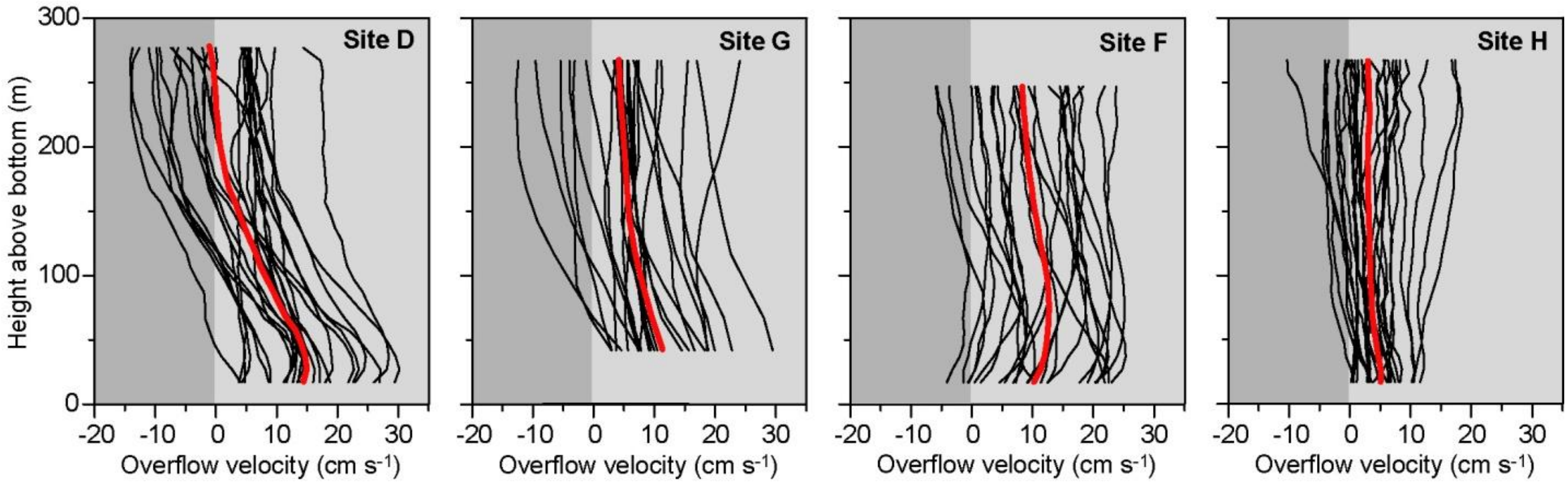

Supplementary Fig. 5. Velocity profiles at Acoustic Doppler Current Profiler sites. 14-day averaged velocity profiles along the overflow direction (Table 1) for Site D, G, F and H (Fig. 1). The background shading indicates velocities towards the Iceland Basin in light shading or towards the Norwegian Sea in dark shading. The red curves show the velocity profiles averaged over the whole deployments.

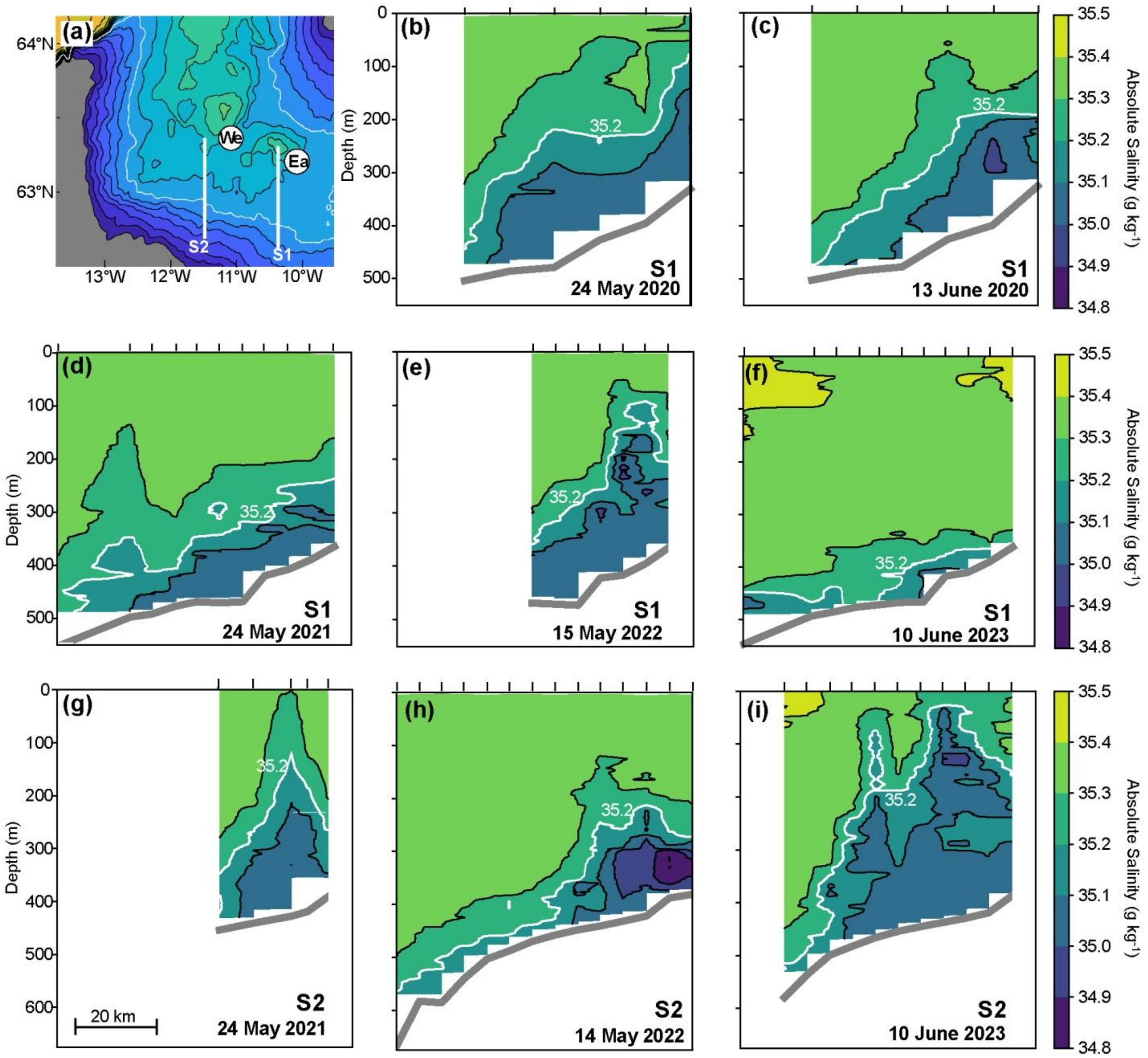

Supplementary Fig. 6. Hydrographic sections on the southwestern flank of the Iceland-Faroe Ridge. Absolute Salinity on repeated occupations of two hydrographic sections, S1 and S2, shown on the map in panel (a). Panels (b) – (f) show results from five occupations of S1. Panels (g) – (i) show three occupations of S2. All the section plots have the same scale and colour coding for Absolute Salinity. Isohalines are shown for every 0.1 g kg^{-1} and the 35.2 g kg^{-1} isohaline enhanced in white. All the section plots have the same vertical (depth) and horizontal (shown in panel (g)) scales.
