# Supplementary material for: Iceland-Faroe overflow revealed by in-situ observations
Source: Nat Commun. 2026 Aug 27;17:10257. doi: 10.1038/s41467-026-77220-w (PMC13616910; doi:10.1038/s41467-026-77220-w)
Supplement: Supplementary file 2 [file 41467_2026_77220_MOESM2_ESM.pdf]

nature portfolio

Peer Review File

Iceland-Faroe overflow revealed by in-situ observations

Corresponding Author: Dr Karin Margretha Larsen

This file contains all reviewer reports in order by version, followed by all author rebuttals in order by version.

Version 0:

Reviewer comments:

Reviewer #1

(Remarks to the Author)
General comments
This manuscript attempts to answer the evasive question of where on the complicated Iceland-Faroe ridge the overflow waters cross into the Atlantic.
The fieldwork done in this study is very extensive, the analysis is robust and the results are definitely interesting to the field. As such, it would be worthy of publication in this journal, but the manuscript could be improved in readability and visualization of the data.

Line comments
Line 47. typo, overflow
Line 48. Where is the occupied section located?
Line 49. “although variably” variable in what aspect?
Line 53. “should be noted” why should it be noted?
Line 55. “there are still many open questions”. The next paragraphs down to line 101 reads like the questions were solved many years ago. As such, it is not clear what is the remaining open question at this point in time are until you get to the very last paragraph (lines 103-109). Even though this review of what has been done is very interesting, I think it could be clearer if it was condensed to the main points. Now it reads like a history lessons of fieldwork more then statements of what we know and what we don't know.
Line 89 (ref 10), make references consistent
Figure 2.
Are PVDs are good choice? It is helpful only in the cases where velocity changes direction during the record, which is only true for a few of the locations. In general, PVDs are not very accessible to a general audience as they are often interpreted like a lagrangian path, but in these cases the actual path of the water is not actually along these line, but will turn towards the west along topography on the south side of the ridge. The rose diagrams in the supplementary are actually easier to interpret.
The depths indicated in the figures suggests that measurements in panel a are shallower than in panel b, which they are not. I think the difference between 17, 18 of 20m above the bottom is not super relevant and the now the values are confusing. What not put the depth relative to the sea surface there?
Line 139. What is 100% validated?
Line 151. Do you mean no volume transport estimate of a water mass denser or colder than a certain threshold is possible? For a publication of this type there should at least be an attempt made to estimate it.
Line 185. It is not so interesting to know what the median temperature is and I’m not sure what the purpose of Fig 5 is. What would be helpful to strengthen the argument of overflow pathways is to know if stronger overflow velocities correspond to lower temperatures. Could this be visualized?
Lines 199-203. This description is very vague/not very concrete. Possible switching of location of the flow is mentioned much later in the manuscript but would be helpful here. Complete with the statement on significance.
Figure 7 is a useful figure to explain what an overflow looks like and how spread out it might be over this region. Would suggest moving this figure up, as Figure 2 possibly.
Line 290. In fact, it doesn’t need to go all the way down in order to join the AMOC. It just needs to flow southward at this density. A lot of it also doesn’t go “all the way” down to the bottom of the basin, stays at intermediate levels as Icelandic Slope Water for example.
Line 295. Yes, this is the Icelandic Slope Water.

Line 314. Why eastward and not northeastward?
Line 325. Stronger overflow?
Line 401. So it is the sea level anomaly anomaly? Why is this done? The distance between the grid points and the ADCP locations does not change, why is it necessary to remove this temporal variability. Is there a paper that described this in more detail and shows that it works?

Data availability "The in-situ datasets utilised in the current study are available from the corresponding author on reasonable request." Data should be freely accessible at the latest at publication.

Reviewer #2

(Remarks to the Author)
Iceland-Faroe overflow revealed by in-situ observations
by Karin Margretha H. Larsen, Gudrid Eriksdottir, Bogi Hansen, Hjalmar Hatun, Steffen M. Olsen, and Svein Osterhus

Larsen et al present a newly-compiled set of results from a number of current meter installations and hydrographic sections aimed at characterizing the portion of the overflow of dense water from the Nordic Seas (ultimately forming North Atlantic Deep Water) that passes between Iceland and the Faroe Islands.

The manuscript combines previously published measurements with new observations, including five recently occupied mooring sites (DEFGH, 2020–2023) and four earlier sites (ABCW, 2003-2017). The datasets are analyzed through a variety of descriptive and statistical diagnostics, including progressive vector diagrams, mean velocity profiles, histograms of bottom velocity and temperature, timeseries of selected variables, and correlations among bottom temperature, bottom velocity, upper-layer velocity, and sea surface height from satellite altimetry.

The results emphasize that the Iceland-Faroes Ridge (IFR) overflow is spatially heterogeneous and variable in time. The spatial distribution appears to be influenced by a combination of the complex ridge topography of banks and channels, the mean upstream isopycnal slope (higher toward Iceland and lower toward the Faroes), and the location and variability of the main pathways of upper-layer Atlantic inflow (including jets and retroflections). The manuscript describes a spatial pattern in which overflow is partially "blocked" near the ends of the ridge by strong upper-layer inflow, while overflow is more prevalent in the central portion where the upper-layer flow retroflects. In addition, there is spatial variability in the temporal relationship (correlation) between the overflow and the upper-layer inflow.

Overall, the manuscript is well written and figures are of high quality. While much of the material presented is clearly motivated and adds valuable information about the geography and variability of the overflow, some aspects of the data description and analysis are underdeveloped.

Strengths: The most compelling aspects of the manuscript are the presentation of new data and the descriptive analysis of the full set of ridge-spanning measurements. The addition of five new mooring sites between 2020 and 2023 substantially expands the observational coverage of the IFR. The authors provide a consistent set of diagnostics across 9 sites, including progressive vector diagrams, mean velocity profiles, and histograms of bottom velocity and temperature, allowing the reader to compare conditions where the dense overflow is relatively strong, weak, intermediate, or absent. Among these analyses, the progressive vector diagrams are particularly informative in illustrating the persistence and directionality of the flow at each location. The velocity and temperature histograms also provide useful qualitative insight into the variability of the bottom layer. The manuscript also compiles relevant prior observations and literature on the IFR system, and the reference list provides a helpful overview of previous studies of this region.

Weaknesses: Several components of the manuscript are not fully integrated or motivated. This includes the correlation analysis, altimetry results, hydrographic sections, physical interpretation, and the implications of the findings for the meridional overturning.

1) The statistical correlation analyses of bottom temperature, bottom velocity, upper-layer velocity, and altimeter sea surface height are not fully motivated by underlying goals. The manuscript provides statistical zero-significance thresholds but does not explain the dynamical meaning of the correlation values themselves. Also, there's a mention of removing the effects of serial correlation (autocorrelation) using a method developed for fish population analysis but not why this modification is needed for physical variables like temperature, surface height, and velocity. As presented, the correlations function mainly as qualitative indicators of the relative strength of the relationship between overflow and inflow. In order to achieve meaning from the results, it might be necessary to move beyond bulk correlation coefficients to a coherence analysis that describes the relationship at different frequencies (including tidal bands, if possible).

2) The altimeter analysis is based primarily on sea surface height time series at the mooring locations. While surface height may be a decent proxy for upper-layer thickness, the surface geostrophic current from surface slope should be a better indicator of upper-layer flow. This could at least be tested by comparison with the upper-layer mooring velocities. If altimetry is successful in providing upper-layer (inflow) velocity at each mooring, it could also support analysis of inflow pathways and timeseries of integrated inflow transport (across the entire ridge or in the particular regions where overflow-blocking is suggested). Particularly interesting would be to construct maps of surface flow patterns associated with, say, periods of stronger or weaker inflow or overflow at the moorings (including the bottom-layer direction shifts at A and C).

3) Although the authors correctly note the difficulty of estimating transport from individual current-meter moorings, the absence of any transport estimates is somewhat surprising given the importance of transport in the broader context of Nordic Seas overflow. Even approximate estimates (with clearly-described assumptions) would help connect the observations to their larger oceanographic significance. One important aspect of this to keep in mind is that, given the tendency of currents to follow topographic contours, speeds may increase in regions of steeper slope due to confluence without an accompanying increase in transport. Also, for the purposes of estimating overflow properties (including transport) more use could be made of the co-located temperature and velocity measurements (e.g., differentiating between the mean direction and magnitude of cold water vs. warm water).

4) The hydrographic sections shown in the manuscript provide valuable information but are not analyzed quantitatively, and only the along-ridge section is integrated into the broader discussion (on spatial inhomogeneity). Points that could be made better from the sections include (a) the range of variability (both temporal and across the sections) in overflow properties, including layer thickness, stratification, cross-sectional area, integrated density anomaly, and transport, (b) the relationship between bottom temperatures as measured by the moorings and these overflow properties, and (c) the relationship between overflow layer thickness and the presence of overlying low-S water.

5) The analysis relies heavily on measurements from the bottom and top ADCP bins and on bottom temperature as proxies for the overflow and inflow layers. While this is understandable given the available instrumentation, the limitations of these proxies should be discussed and evaluated, using more robust or complete metrics (e.g., the hydrographic sections, integrated layer transports, etc). For example, bottom temperature may represent either a thin overflow layer or a deep column of dense water.

Additional areas for improvement or further development:

6) Of the plots presented in the supplement, the one showing the mostly barotropic nature of the current variability (Fig.s2) is probably the most useful. The photos of the mooring equipment are certainly interesting, although perhaps not essential. Without additional discusssion and integration, it's less clear what the need is for the additional figures and tables showing sections, timeseries, and vertical correlation structure.

7) The histograms of mooring velocity and bottom temperature are qualitatively informative in their present form, but the discussion could be better developed. The velocity distributions appear quite similar to each other and not too far from a symmetric Gaussian shape, though with variation in width (variance) and central tendancy (mean). However, the bottom temperature distributions show a larger range of variability, including skewness and secondary modes. Could these perhaps represent a superposition of 2 distrubutions--a narrow cold overflow mode and a broad spread of inflow temperatures? One potential avenue of investigation would be to fit parameters for these 2 distributions (e.g., a narrow and a broad Gaussian) to each site. This would give a metric for the relative prevalance of overflow and inflow water (though only at the bottom).

8) Tidal variability is not described in the manuscript because it is "removed" using harmonic analysis before constructing the 1-day averages. However, if velocity and thickness (or density) are correlated at diurnal and/or semidiurnal periods, there could be a significant tidal component to overflow dense water transport.

9) A similar current meter deployment at site I (fig. 1) is mentioned but not included in the analysis of the mooring records presented. If these data are still available, it seems that site I would be worth treating with the same set of analyses as the 9 sites that are in the manuscript. At present, it's a bit puzzling why this site is mentioned as an important part of IFR overflow research but not included in the analysis.

10) There are hints in the data presented that the dense overflow and upper-layer inflow could be characterized by particular dynamical modes or states, including: (a) the coordinated switch between westward and eastward near-bottom flow at sites A and C (Fig.2), (b) the presence or absence of the intermediate low-S watermass between the dense overflow and the warm Atlantic water, (fig 7b), (c) bottom temperature transitions between cold and warm water at multiple sites. Although not currently presented in this way, this could be a useful unifying theme for the manuscript. A challenge is that each geographic location seems to have it's own unique temporal pattern.

11) Although climate change and long-term variability are mentioned briefly in the abstract (in the sense that the IFR overflow may be more sensitive to climate changes than other Nordic seas dense water overflows), this topic is not really explored in the analysis. If true, this sensitivity could result from either changes to the dense water supply or through the interactions with a changing upper-layer Atlantic inflow. Given the claims that the IFR overflow is influenced by the upper-layer inflow at some places along the ridge more than others, this seems like a hypothesis that could be subjected to some initial testing using the methods in this manuscript--possibly extended using the altimeter record. On the other hand, the possibility of interannual variations in the dense water supply could be seen to invalidate the principal approach taken in the manuscript to develop a geographical picture of the overflow using measurements from different years. This very real drawback is also not addressed, but should at least be mentioned.

12) One final possibility may be worth discussing, based on what is presented in the manuscript: Since the ADCP moorings at sites H and F show a fairly barotropic velocity structure with very little overflow layer shear, is it possible that they are evidence of full-depth anticyclonic "Taylor cap" circulation around the neighboring banks (high points) on the ridge and not pathways for significant amounts of dense water that descends into the Iceland Basin? This may be even more likely at site H, where the bottom temperature distribution doesn't seem to show any sign of the cold overflow mode (Fig.5h). The PVDs

in Fig.2 do show some veering between the upper and lower layers, so this could indicate some down-slope Ekman transport at the bottom. But since one of the few conclusions of the paper is that much of the dense IFR overflow occurs at the sites in the middle of the ridge, this seems to suggest that sites H and F (the western pathway) could instead have minimal contributions.

Reviewer #3

(Remarks to the Author)
Dear Authors, and Editors,

Thank you for the opportunity to review this well-written and insightful paper.

Based on nine almost year-long data records, this study provides valuable new insight about the configuration of overflow pathways across the Iceland-Faroe ridge. The analyses are based upon quality-checked, de-tided, daily average current velocity data recorded by ADCPs, supported by hydrographic data (mainly temperature) from CTDs and loggers and complemented by gridded Sea Level Anomaly data. The overflow indicators used are near-bottom velocity and bottom temperature.

The analyses reveal a strong and persistent overflow in the central parts of the ridge, with weaker signals on the 'sides', and the "Western" and "Eastern" pathways are identified as the major pathways for the overflow. Temporal variability on daily and bi-weekly time scales show a pulsating flow at these sites. Owing to the nature of the monitoring program (single ADCPs at each site) volume transport estimates are not yet possible. Relationships between overflow indicators (velocity, temperature) and two potential predictors (AW inflow strength, sea level anomaly) show several strong correlations, although the underlying mechanisms remain to be fully resolved.

The manuscript clearly guides the reader through both similarities and differences across the investigated sites with well-structured descriptions of the background, methods, analyses, figures, and discussion. The statistical treatment is solid with clear reporting of significance levels and the conclusions are consistent with the evidence presented. I appreciate both the careful analysis of this massive dataset, the added value of the snapshot CTD sections and also the supplemental information. The interpretations are sensible, and the broader context is well articulated.

Overall, this study represents excellent progress in deciphering the flow structure across the ridge, reducing several long-standing uncertainties for this overflow region. I look forward to future monitoring efforts in this area, which will hopefully allow overflow transport estimates and further insights into the controlling mechanisms.

I have no major issues or questions but include a few comments and suggestions of various scope, intended to further improve the manuscript. I recommend acceptance after minor revision.

- - - Comments/suggestions for the text, by line number:

L72: Consider rephrasing from "…and no week during these two years had an average core velocity less than 30 cm s-1" to e.g. "…and throughout the entire record the weekly average core velocity remained above 30 cm s-1".

L90: 'Should 'had' rather be 'has', and 'remaining' be 'remain' or 'remained'?

L91: Simplify the "is joining in a…" to "According to Perkins et al.6, this water joins a focused flow over the northwestern part of the ridge and is transported towards the exit of the Western Valley (…).", which I think is what you mean.

L108: CTD not defined/spelled out earlier. Could write hydrographic observations here if it fits better with a definition on L150?

L127: Nice approach to use PVDs normalized by deployment duration although I'm confused about the resulting velocities being called 'residual velocities'? In a sense correct since they are based on de-tided velocities but confusing the context of PVDs. Aren't these velocities 'record-mean velocities', to be compared to/guided by the velocity scale shown? I must confess I have no good suggestion of a perfect term to use instead but please give this a thought and possibly adjust terminology.

L165: "Overflow persistence" For me, persistence would also include the duration of an overflow event (e.g. the minimum number of consecutive days of such events?). At least for sites with quite normal distribution between inflow and outflow. (For the ones with high %, I guess it's already sign enough for a persistent overflow. Have you looked into that?

L185: "…bottom temperature was at or below 2oC at least half of the time, indicating overflow water." I take it you mean temperature at or below 2oC indicates overflow water, and not the "half of the time"? It would be good to support the 2oC with a reference.

L248: In the caption call (a)-(i) panels and not figure.

L256: What is meant with '…almost complete coverage'. Is it the observations, or rather the number/network of instruments that could have been more complete?

L258: "With that caveat, the observations provide a consistent picture." Do you mean Despite that caveat?

L369: Had to read twice (and check SupplTable1): clarify by rephrasing to e.g. "All ADCPs sampled at 20 min intervals, with one ping per ensemble for the BB ADCP and 12 pings per ensemble for the LR ADCP."

L373: I find a reference to the tidal analysis is lacking (is the analysis made by in-house scripts/routines?). Also, to guide the reader I would start with "To de-tide the velocities, a harmonic analysis is…"

L379: How often did the SBE37/16 sample temperature?

L392: If not in the text (cf. L108, L150), spell out CTD here.

L393: SeaBird without dash

L393-394: Perhaps use 'were' instead of 'are'?

L399: Consider rephrasing from "For each of the ADCP sites, the closest grid point was found and daily SLA-values selected." to 'For each of the ADCP sites, daily SLA-values were extracted from the closest grid point. The distance between…'

L402: How was this extent determined? Just to fit the region properly, or by any other restrictions/features?

L414: Just for clarity, one could add the scripting language/version used for the analysis?

- - - Comments/suggestions on the figures:

Figure 2: The PVDs, that are the most important and interesting features in the plot, don't stand out sufficiently and are easily confused with labels and the 500m-isobaths, at least when printed on paper (in digital format it is better since easier to magnify.) I wonder if black PVDs would be easier to follow, after all? And the bin-depth labels can be put in white textboxes. (One part of me would prefer seeing this on grey scale topography, as for Figure 1, which I think would make the important features stand out better.)
Also, although you justify it in the captions, I find it hard when scales do not match between maps one is supposed to compare. Could both maps be drawn on the same scale after all?

Figures 3, 4, and 5: Skip the panel labels (a, b, c, …). These are redundant and confusing in relation to the Site Letters, which are what you generally refer to in the text (and very well so). (Increase font size of the Site letter somewhat.)

Figure 6: Make panel (c) for the same temperature range as panel (a), for easier comparison?

Figure 7: nice 'snapshot' of a typical overflow!

Figure 8 (and Suppl.Figure 7): I was about to ask why the vertical and horizontal scales had to differ for the two transects (as stated in the caption). But it looks as if all panels indeed are on the vertical and horizontal same scale but not over the same ranges over depth and distance (since at different locations). Is that what you mean?
Also, it would be helpful with indications of the CTD station locations, as in Figure 7 and
Suppl.Figure 4.

Suppl.Figure 4: Why different horizontal and vertical scales? It would be helpful to keep constant scales (although the ranges may vary for each site, cf. comment for Figure 8).

Suppl.Figure 6: In the caption, I think 'preferred' is a better word than 'selected': "…site E where no specific overflow direction seems to be selected." (BTW: Intriguing with such different results for relatively close site locations (and deployment periods!)

Version 1:

Reviewer comments:

Reviewer #2

(Remarks to the Author)
Iceland-Faroe overflow revealed by in-situ observations
by Karin Margretha H. Larsen, Gudrid Eriksdottir, Bogi Hansen, Hjalmar Hatun, Steffen M. Olsen, and Svein Osterhus

The revised manuscript has been much improved by both the additions and removals of figures and text. In particular, the scatter plots in Fig. 5 are a nice addition. I have only a few minor comments below and hope to see this paper published very soon!

l.90 This sentence doesn't quite make sense as written and could be fixed in a few different ways. One possibility is to (a) substitute "combined" for "joint" and (b) add "is" after "water".

l.241 Change "Fig. 6" to "Fig. 7"

l.260-261 "transport per unit width" is a clearer label than "transport density". I'd suggest sticking with the former.

l.288-291 Since the Eastern and Western pathways seem to be somewhat anti-correlated, it's likely that the summed transport of the two is a more stable value which could be stated with greater confidence.

l.324-325 Isn't it more common to state the p<0.05 result as "different from zero with 95% confidence", rather than "at the 5% level"? Same for 99% and 99.9%. That would make more sense to me (though I suspect readers can figure it out).

Reviewer #3

(Remarks to the Author)
Dear Authors, and Editors,

Thank you to the Authours for the thorough revision of your manuscript and clear response back to all reviewers. I find you have solidly implemented the comments by us all (or, in a few cases, provided well motivated justifications when keeping things 'as is'), and arrived at more solid and well-balanced presentation of your valuable study.

I particularly value the re-worked section "Controlling mechanisms", the move of the original Fig7 to now Fig2, the inclusion of Table 1 with ADCP characteristics plus estimates, and the new Fig5 presenting combined numberd from the original Figures 4 & 5 (and I hope the original histograms in fig5 can be revisited in upcoming analyses?).

I also appreciate the work on arriving at the transport estimates (or at least as-close-to-transport-estimates-as-possible) for the main branches, as requested by R1 and R2. I think the authors do a good job motivating their approach and choices for this analysis, and also being clear about the related (high) uncertainties. Hopefully, future monitoring will provide more secure estimates.

The additions and revisions to the text, sections/headers related to the above modifications are satisfactory, addressing the comments from all reviewers. Also smaller changes, such as the redistribution of the panels/sites in the current Figs4 and 5, has improved the presentation.

For my part, I recommend the manuscript to be accepted for publication, subject to only a few technical corrections (cf. the below comments).

I noted down a few things that could be worth a check, for improved readability,

Manuscript:

L61 "The most perplexing unresolved question, however, ..." ?

(L70 mistyping around the Beaird9 ref, L74+L75 reformat cm s-1)

L135 A bit convoluted. Sufficient with: "Here, we use daily averaged velocities measured by the ADCPs at several levels ("bins") above the instrument."?

L144 In fig3 caption, simplify to: "The orange traces show PVDs based on daily averaged velocities for (a) the deepest bin, Bin-1, and (b) the Top-bin (see text and Methods). At the end of each PVD is shown the centre depth for (a) Bin-1 and (b) the Top-bin. "

L170 "Main characteristics for the seven sites are listed in Table 1, which illustrates the dominance of the three main overflow sites: ...". Not entirely evident (from the table readings) which properties you define the dominance by (e.g. "with regard to average velocity...")

L328 "The bottom four rows..." or "The last four rows..."?

The Supplement:
Supplementary Table 1. Since the ADCPs are all moored deep it is perhaps evident, but could be good to clarify that all ADCPs were "upward looking". This would also aid the bin-explanation in L135-138. May also be invoked in L400: "The

upward looking ADCPs were either placed...".

Supplementary Fig. 5. Is it possible to include the average profiles form Fig.4 here (e.g. as thick white line?), as aid to the reader since both the x- and y-ranges have changed?

Open Access This Peer Review File is licensed under a Creative Commons Attribution 4.0 International License, which permits use, sharing, adaptation, distribution and reproduction in any medium or format, as long as you give appropriate credit to the original author(s) and the source, provide a link to the Creative Commons license, and indicate if changes were made.
In cases where reviewers are anonymous, credit should be given to 'Anonymous Referee' and the source.
The images or other third party material in this Peer Review File are included in the article’s Creative Commons license, unless indicated otherwise in a credit line to the material. If material is not included in the article’s Creative Commons license and your intended use is not permitted by statutory regulation or exceeds the permitted use, you will need to obtain permission directly from the copyright holder.
To view a copy of this license, visit https://creativecommons.org/licenses/by/4.0/

RESPONSE TO REVIEWER COMMENTS

We thank all three reviewers for very positive and constructive comments. As requested from the editor, we have made a substantial revision of the manuscript. We have tried to respond to as many of your suggestions as possible, while still keeping the word limit of the journal. In a few cases the reviewers had opposite opinions and here we have tried to argue for what we considered was the most appropriate choice for the storyline in the manuscript. The main changes in the revised manuscript are listed here:

- A part of the Introduction has been shortened and rewritten
- Figure 7 (Hydrographic section along the IFR crest) has been moved forward, such that it now is Figure 2.
- Figure 4 (histogram of overflow velocity) and 5 (histogram of bottom temperature) have been removed and a new Figure 5 is introduced, showing Overflow velocity against bottom temperature.
- A new table has been introduced, showing ADCP characteristics, incl. Overflow stability and Transport density estimates.
- Parts of the text in the Discussion has been rewritten and moved forward to Results, where also a new section on Overflow volume transport has been added. Here we provide estimates of overflow transport at the Eastern and Western pathways, although with very high uncertainties.

As an additional comment, we did already in the preparation of the original manuscript investigate some of the suggestions for further analysis suggested by the referees, but without finding any results that seriously affected our main conclusions. They were therefore omitted, partly to stay within the required maximum length of the manuscript, but also because including them, in our opinion, would have reduced the readability of the manuscript.

In the revised manuscript, changes are in red font. Below we reply to the individual comments, also in red.

Reviewer #1 (Remarks to the Author):

General comments

This manuscript attempts to answer the evasive question of where on the complicated Iceland-Faroe ridge the overflow waters cross into the Atlantic.

The fieldwork done in this study is very extensive, the analysis is robust and the results are definitely interesting to the field. As such, it would be worthy of publication in this journal, but the manuscript could be improved in readability and visualization of the data.
Thank you for your comments. We have tried to respond to all your comments as well as possible.

Line comments

Line 47. typo, overflow
Corrected.

Line 48. Where is the occupied section located?
The section was occupied along the southwestern flank. Text has been added to describe the location.

Line 49. “although variably” variable in what aspect?
In both time and space. Text has been added to clarify.

Line 53. “should be noted” why should it be noted?
Good point. The sentence has been rephrased.

Line 55. “there are still many open questions”. The next paragraphs down to line 101 reads like the questions were solved many years ago. As such, it is not clear what is the remaining open question at this point in time are until you get to the very last paragraph (lines 103-109). Even though this review of what has been done is very interesting, I think it could be clearer if it was condensed to the main points. Now it reads like a history lessons of fieldwork more then statements of what we know and what we don't know.
Thank you for the comment. We agree and have now tried to rewrite these paragraphs leaving out unnecessary information.

Line 89 (ref 10), make references consistent
Of course. Many of the references mentioned directly in the text have been removed in the rewriting of the text and we now follow, what is usually used in Nature Communications papers (e.g. like “According to Herman[10], …”)

Figure 2. Are PVDs are good choice? It is helpful only in the cases where velocity changes direction during the record, which is only true for a few of the locations. In general, PVDs are not very accessible to a general audience as they are often interpreted like a lagrangian path, but in these cases the actual path of the water is not actually along these line, but will turn towards the west along topography on the south side of the ridge. The rose diagrams in the supplementary are actually easier to interpret.

The depths indicated in the figures suggests that measurements in panel a are shallower than in panel b, which they are not. I think the difference between 17, 18 of 20m above the bottom is not super relevant and the now the values are confusing. What not put the depth relative to the sea surface there?
We agree that PVDs may be misinterpreted as lagrangian paths and we have added a sentence to hopefully prevent that. We considered using rose diagrams instead of PVDs, but adding them to the map would have made them overlap one another, especially for sites D, G and E. Since both R2 and R3 seemed to be happy with the PVDs, we decided to keep them, but Figure 2 has been modified as suggested by R3. We have also followed your recommendation and Figure 2a now shows the depth relative to sea surface rather than height above bottom.

Line 139. What is 100% validated?
This was badly phrased. The definition of the “Top-bin” has been moved and the text modified to a more correct phrasing.

Line 151. Do you mean no volume transport estimate of a water mass denser or colder than a certain threshold is possible? For a publication of this type there should at least be an attempt made to estimate it.
Following yours and R2’s suggestion, we have tried to calculate estimates of the volume transport based on ADCP data and rough estimates of the height and width of the flow. This is included in a new section within the Results part. The reason for not including this in the original version was that the uncertainties were so large. This is also clear from the new text where we emphasize that *“the two main pathways, together with smaller contributions from the other locations may well combine to a total IFR-overflow transport approaching 1 Sv, especially if entrainment downstream of the ridge crest is included”*.

Line 185. It is not so interesting to know what the median temperature is and I'm not sure what the purpose of Fig 5 is. What would be helpful to strengthen the argument of overflow pathways is to know if stronger overflow velocities correspond to lower temperatures. Could this be visualized?
Thank you for this comment. We have followed your suggestion and introduced a new figure (Figure 5). This also made the old Figures 4 and 5 more or less redundant and so they have been deleted.

Lines 199-203. This description is very vague/not very concrete. Possible switching of location of the flow is mentioned much later in the manuscript but would be helpful here. Complete with the statement on significance.
We have added some explanatory text and tried to follow your recommendation.

Figure 7 is a useful figure to explain what an overflow looks like and how spread out it might be over this region. Would suggest moving this figure up, as Figure 2 possibly.
We have followed your suggestion – Figure 7 has been moved forward and is now Figure 2.

Line 290. In fact, it doesn't need to go all the way down in order to join the AMOC. It just needs to flow southward at this density. A lot of it also doesn't go "all the way" down to the bottom of the basin, stays at intermediate levels as Icelandic Slope Water for example.
We agree. This was badly phrased in the original version. We have modified the text.

Line 295. Yes, this is the Icelandic Slope Water.
Yes, we agree. We have added Johns et al (new ref 13), who discuss Icelandic Slope Water in their work.

Line 314. Why eastward and not northeastward?
We tried various directions and found the eastward to give the most consistent results. The text has been modified to indicate this.

Line 325. Stronger overflow?
Corrected.

Line 401. So it is the sea level anomaly anomaly? Why is this done? The distance between the grid points and the ADCP locations does not change, why is it necessary to remove this temporal variability. Is there a paper that described this in more detail and shows that it works?
This was badly phrased. We have now modified the text to emphasize that the "correction" was made to focus on mesoscale rather than large-scale sea level variations. We have added that this procedure also increased the magnitudes of the correlation coefficients, which we checked.

Data availability "The in-situ datasets utilised in the current study are available from the corresponding author on reasonable request." Data should be freely accessible at the latest at publication.
Certainly. This was also our intention. The data are available as ASCII files at www.envofar.fo/index.php?page=climate.

Reviewer #2 (Remarks to the Author):

Iceland-Faroe overflow revealed by in-situ observations

by Karin Margretha H. Larsen, Gudrid Eriksdottir, Bogi Hansen, Hjalmar Hatun, Steffen M. Olsen, and Svein Osterhus

Larsen et al present a newly-compiled set of results from a number of current meter installations and hydrographic sections aimed at characterizing the portion of the overflow of dense water from the Nordic Seas (ultimately forming North Atlantic Deep Water) that passes between Iceland and the Faroe Islands.

The manuscript combines previously published measurements with new observations, including five recently occupied mooring sites (DEFGH, 2020–2023) and four earlier sites (ABCW, 2003-2017). The datasets are analyzed through a variety of descriptive and statistical diagnostics, including progressive vector diagrams, mean velocity profiles, histograms of bottom velocity and temperature, timeseries of selected variables, and correlations among bottom temperature, bottom velocity, upper-layer velocity, and sea surface height from satellite altimetry.

The results emphasize that the Iceland-Faroes Ridge (IFR) overflow is spatially heterogeneous and variable in time. The spatial distribution appears to be influenced by a combination of the complex ridge topography of banks and channels, the mean upstream isopycnal slope (higher toward Iceland and lower toward the Faroes), and the location and variability of the main pathways of upper-layer Atlantic inflow (including jets and retroflections). The manuscript describes a spatial pattern in which overflow is partially "blocked" near the ends of the ridge by strong upper-layer inflow, while overflow is more prevalent in the central portion where the upper-layer flow retroflects. In addition, there is spatial variability in the temporal relationship (correlation) between the overflow and the upper-layer inflow.

Overall, the manuscript is well written and figures are of high quality. While much of the material presented is clearly motivated and adds valuable information about the geography and variability of the overflow, some aspects of the data description and analysis are underdeveloped.

Strengths: The most compelling aspects of the manuscript are the presentation of new data and the descriptive analysis of the full set of ridge-spanning measurements. The addition of five new mooring sites between 2020 and 2023 substantially expands the observational coverage of the IFR. The authors provide a consistent set of diagnostics across 9 sites, including progressive vector diagrams, mean velocity profiles, and histograms of bottom velocity and temperature, allowing the reader to compare conditions where the dense overflow is relatively strong, weak, intermediate, or absent. Among these analyses, the progressive vector diagrams are particularly informative in illustrating the persistence and directionality of the flow at each location. The velocity and temperature histograms also provide useful qualitative insight into the variability of the bottom layer. The manuscript also compiles relevant prior observations and literature on the IFR system, and the reference list provides a helpful overview of previous studies of this region.

Weaknesses: Several components of the manuscript are not fully integrated or motivated. This includes the correlation analysis, altimetry results, hydrographic sections, physical interpretation, and the implications of the findings for the meridional overturning.

We thank you for your thorough and constructive comments. In addition to general comments, you have provided 12 suggestions on how we can improve the manuscript. Given the word limit of the Journal, it was not possible to incorporate them all, but we have included as many as possible in order to improve the manuscript. Below, we reply to your suggestions and try to argue for those who were not incorporated.

1) The statistical correlation analyses of bottom temperature, bottom velocity, upper-layer velocity, and altimeter sea surface height are not fully motivated by underlying goals. The manuscript provides statistical zero-significance thresholds but does not explain the dynamical meaning of the

correlation values themselves. Also, there's a mention of removing the effects of serial correlation (autocorrelation) using a method developed for fish population analysis but not why this modification is needed for physical variables like temperature, surface height, and velocity. As presented, the correlations function mainly as qualitative indicators of the relative strength of the relationship between overflow and inflow. In order to achieve meaning from the results, it might be necessary to move beyond bulk correlation coefficients to a coherence analysis that describes the relationship at different frequencies (including tidal bands, if possible).
We agree that correlation analysis has limited explanatory power. In preparing the original manuscript, we did explore other methods, but we did not find any new results that would affect the main conclusions. Since the length of the manuscript already was (and still is) close to the limits set by the journal, information on these methods and results was omitted.

We still feel that the correlation analyses do provide important information, but we also agree that our motivation for these analyses and their interpretations not were well described. This was especially the case for the section "Controlling mechanisms", which has been rewritten.

As to the method used for estimating statistical significance, serial correlation in time series may introduce unrealistic impressions of statistical significance in correlation analysis since it decreases the effective number of degrees of freedom. There are various ways to correct for this (e.g., von Storch, 1999, https://doi.org/10.1007/978-3-662-03744-7_2). The method we used was, as noted by R2, originally developed for analysis of fish populations, but we have found it satisfactory also for more physical time series (e.g., Hansen et al., 1918 (ref 14); 2023 (ref 15)).

2) The altimeter analysis is based primarily on sea surface height time series at the mooring locations. While surface height may be a decent proxy for upper-layer thickness, the surface geostrophic current from surface slope should be a better indicator of upper-layer flow. This could at least be tested by comparison with the upper-layer mooring velocities. If altimetry is successful in providing upper-layer (inflow) velocity at each mooring, it could also support analysis of inflow pathways and timeseries of integrated inflow transport (across the entire ridge or in the particular regions where overflow-blocking is suggested). Particularly interesting would be to construct maps of surface flow patterns associated with, say, periods of stronger or weaker inflow or overflow at the moorings (including the bottom-layer direction shifts at A and C).
We do agree that the surface geostrophic current from surface slope is a better indicator of upper layer flow than sea surface height by itself and we do find fair agreement (correlation) with upper layer mooring velocities. Some of these results were published in Hansen et al. (2023) (Ref 15). For comparison with overflow, we feel, however, that it is better to use the upper layer mooring velocities themselves rather than the geostrophic surface current, which is limited by the spatial resolution of the altimetry data. Therefore, we have used our observed values for U_T in Table 2. Our motivation for using sea surface height (and not slope) was that it might be a good indicator for upper-layer thickness, as noted by R2, since then it would also be an indicator for the height of the overflow layer. As previously noted, we realize that the text describing our motivation an interpretation was not very clear. Hopefully, the new version is better.

3) Although the authors correctly note the difficulty of estimating transport from individual current-meter moorings, the absence of any transport estimates is somewhat surprising given the importance of transport in the broader context of Nordic Seas overflow. Even approximate estimates (with clearly-described assumptions) would help connect the observations to their larger oceanographic significance. One important aspect of this to keep in mind is that, given the tendency of currents to follow topographic contours, speeds may increase in regions of steeper slope due to confluence without an accompanying increase in transport. Also, for the purposes of estimating

overflow properties (including transport) more use could be made of the co-located temperature and velocity measurements (e.g., differentiating between the mean direction and magnitude of cold water vs. warm water).

As mentioned in response to a similar question from R1, estimates of volume transport were omitted from the original version because limitations by the available data made the estimates very uncertain. We have now provided a new Figure 5, which compares the velocity at the deepest layer with bottom temperature. In a new section in the Results part, we have now included volume transport estimates of overflow water at the two main branches, although they have, as mentioned, very high uncertainties (factor of five).

4) The hydrographic sections shown in the manuscript provide valuable information but are not analyzed quantitatively, and only the along-ridge section is integrated into the broader discussion (on spatial inhomogeneity). Points that could be made better from the sections include (a) the range of variability (both temporal and across the sections) in overflow properties, including layer thickness, stratification, cross-sectional area, integrated density anomaly, and transport, (b) the relationship between bottom temperatures as measured by the moorings and these overflow properties, and (c) the relationship between overflow layer thickness and the presence of overlying low-S water.

We only have a few hydrographic sections available for analysis and these are "scattered" in both time and place. From the bottom temperature records it is seen (Figure 6) that there are large temporal and spatial fluctuations. Given this high variability, it is difficult to give a robust estimate of the variability of the overflow properties based on the hydrographic sections. Nevertheless, we now have used the hydrographic sections to estimate the height of the overflow layer for calculating transports.

5) The analysis relies heavily on measurements from the bottom and top ADCP bins and on bottom temperature as proxies for the overflow and inflow layers. While this is understandable given the available instrumentation, the limitations of these proxies should be discussed and evaluated, using more robust or complete metrics (e.g., the hydrographic sections, integrated layer transports, etc). For example, bottom temperature may represent either a thin overflow layer or a deep column of dense water.

We have now added a section on overflow volume transport where we have tried to follow these suggestions. The results are, however, associated with very high uncertainties, which was our original reason for omitting them.

Additional areas for improvement or further development:

6) Of the plots presented in the supplement, the one showing the mostly barotropic nature of the current variability (Fig.s2) is probably the most useful. The photos of the mooring equipment are certainly interesting, although perhaps not essential. Without additional discusssion and integration, it's less clear what the need is for the additional figures and tables showing sections, timeseries, and vertical correlation structure.

Thank you for the comment on the Supplementary material. We have removed the original Supplementary Table 2, Supplementary Fig. 1 and Supplementary Fig. 5. The Overflow direction in Supplementary Table 1 (last column) has been moved to the new Table 1 in the manuscript. But we have also added a new Supplementary Fig. 1 on the relation between bottom temperature and overflow transport.

7) The histograms of mooring velocity and bottom temperature are qualitatively informative in their present form, but the discussion could be better developed. The velocity distributions appear quite similar to each other and not too far from a symmetric Gaussian shape, though with variation in

width (variance) and central tendancy (mean). However, the bottom temperature distributions show a larger range of variability, including skewness and secondary modes. Could these perhaps represent a superposition of 2 distrubutions--a narrow cold overflow mode and a broad spread of inflow temperatures? One potential avenue of investigation would be to fit parameters for these 2 distributions (e.g., a narrow and a broad Gaussian) to each site. This would give a metric for the relative prevalance of overflow and inflow water (though only at the bottom).
This could be an interesting analysis, but limited to the bottom, as noted. This would also be a quite detailed analysis of overflow characteristics, which is not the main objective of this study. We have therefore not included it here, but it could be implemented in future analysis of IFR overflow.

8) Tidal variability is not described in the manuscript because it is "removed" using harmonic analysis before constructing the 1-day averages. However, if velocity and thickness (or density) are correlated at diurnal and/or semidiurnal periods, there could be a significant tidal component to overflow dense water transport.
The tidal velocities over the IFR are quite strong and may well contribute to the overflow when the tidal current is in the overflow direction. We have not been able to determine, however, how much of that water continues as overflow when the tidal current reverses. We also tried to investigate whether the daily averaged overflow velocity could be linked to the 14-day tidal neap-spring cycle, but without any clear indication of this. We therefore felt that including a discussion on tidal effects would not contribute new information, but rather reduce the overall readability of the manuscript.

9) A similar current meter deployment at site I (fig. 1) is mentioned but not included in the analysis of the mooring records presented. If these data are still available, it seems that site I would be worth treating with the same set of analyses as the 9 sites that are in the manuscript. At present, it's a bit puzzling why this site is mentioned as an important part of IFR overflow research but not included in the analysis.
The main objective of this study was to provide evidence for if there is persistent overflow across the IFR ridge and if the overflow has main branches or is scattered across the whole ridge. The results from the nine ADCP deployments along the ridge have answered these questions and have also shown, that there likely is sufficient overflow volume transport (thank you again for pushing us to estimate the transport, which we actually are quite happy with) to supply the jet at site I and thereby, at least partly, solving that puzzle. Analysis of the ADCP data from site I, similar to those presented here, have already been published in Olsen et al., 2016 (ref. 7) and were also included in a doctoral thesis (ref 12). Since site I is not part of the crest sites and new observations are currently being collected at this site in the EPOC project, further analysis will be performed of the connection between the ridge crest and site I, but depending on data return.

10) There are hints in the data presented that the dense overflow and upper-layer inflow could be characterized by particular dynamical modes or states, including: (a) the coordinated switch between westward and eastward near-bottom flow at sites A and C (Fig.2), (b) the presence or absence of the intermediate low-S watermass between the dense overflow and the warm Atlantic water, (fig 7b), (c) bottom temperature transitions between cold and warm water at multiple sites. Although not currently presented in this way, this could be a useful unifying theme for the manuscript. A challenge is that each geographic location seems to have it's own unique temporal pattern.
Yes, indeed, and while planning the new set of deployments, we had hoped to find indications of such modes or states. For logistical reasons, only a few of the ADCP deployments were concurrent (A and C as well as F and G), which makes it difficult to establish relationships. Figure 6 was an attempt, but it only gives hints. We also tried to use the altimetry data, which cover the whole period of

deployments, to link non-concurrent observations, but again without any statistically significant results. We hope in the future to be able to proceed with these ideas by combining our observations with numerical modelling.

11) Although climate change and long-term variability are mentioned briefly in the abstract (in the sense that the IFR overflow may be more sensitive to climate changes than other Nordic seas dense water overflows), this topic is not really explored in the analysis. If true, this sensitivity could result from either changes to the dense water supply or through the interactions with a changing upper-layer Atlantic inflow. Given the claims that the IFR overflow is influenced by the upper-layer inflow at some places along the ridge more than others, this seems like a hypothesis that could be subjected to some initial testing using the methods in this manuscript--possibly extended using the altimeter record. On the other hand, the possibility of interannual variations in the dense water supply could be seen to invalidate the principal approach taken in the manuscript to develop a geographical picture of the overflow using measurements from different years. This very real drawback is also not addressed, but should at least be mentioned.

We understand your concern. Our argument for the (supposed) special sensitivity of the IFR-overflow was just that it is the shallowest of the four overflow branches, but we have to agree that this is actually pure speculation. Since there has to be a balance between total inflows and total outflows (to/from the Arctic Mediterranean), one could even argue that a potential reduction of the total overflow might lead to a reduced Atlantic inflow, which could mean less blocking of the IFR-overflow. We have to admit that we ought not to have this speculative statement without further discussion, and certainly not in the Abstract. We have therefore removed this sentence from the Abstract.

12) One final possibility may be worth discussing, based on what is presented in the manuscript: Since the ADCP moorings at sites H and F show a fairly barotropic velocity structure with very little overflow layer shear, is it possible that they are evidence of full-depth anticyclonic "Taylor cap" circulation around the neighboring banks (high points) on the ridge and not pathways for significant amounts of dense water that descends into the Iceland Basin? This may be even more likely at site H, where the bottom temperature distribution doesn't seem to show any sign of the cold overflow mode (Fig.5h). The PVDs in Fig.2 do show some veering between the upper and lower layers, so this could indicate some down-slope Ekman transport at the bottom. But since one of the few conclusions of the paper is that much of the dense IFR overflow occurs at the sites in the middle of the ridge, this seems to suggest that sites H and F (the western pathway) could instead have minimal contributions.

Yes, we believe that there is good evidence for an anticyclonic "Taylor cap" over the northern region of the IFR. This was discussed in an earlier publication, see Figure 14 in Hansen et al. (2023), Ref. 15 in the new version of this manuscript. From that study, the center of this Taylor cap would be located north of both H and F. The closed circulation loop may well extend all the way to site H and we make no claim that H is an important overflow contributor, but we find it less likely that the closed loop extends all the way to site F (the Western pathway), at least for the deep parts. Our main argument for that is panel (a) in the new Figure 5, where strong Bin-1 overflow velocity only is seen for low bottom temperatures (see also the new Supplementary Figure 1). The uppermost water over site F may perhaps be part of a closed loop, at least in periods. For the deeper water, we find it likely that it continues flowing along this path as illustrated by the thick blue arrow in Figure 1, but only until the water enters the southwestern parts of the Western valley, after which it turns towards the southwest to join the overflow jet over the south-Icelandic slope (Figure 1). Unfortunately, we believe that only combining our observations with numerical modeling can resolve this.

Reviewer #3 (Remarks to the Author):

Dear Authors, and Editors,

Thank you for the opportunity to review this well-written and insightful paper.

Based on nine almost year-long data records, this study provides valuable new insight about the configuration of overflow pathways across the Iceland-Faroe ridge. The analyses are based upon quality-checked, de-tided, daily average current velocity data recorded by ADCPs, supported by hydrographic data (mainly temperature) from CTDs and loggers and complemented by gridded Sea Level Anomaly data. The overflow indicators used are near-bottom velocity and bottom temperature.

The analyses reveal a strong and persistent overflow in the central parts of the ridge, with weaker signals on the 'sides', and the "Western" and "Eastern" pathways are identified as the major pathways for the overflow. Temporal variability on daily and bi-weekly time scales show a pulsating flow at these sites. Owing to the nature of the monitoring program (single ADCPs at each site) volume transport estimates are not yet possible. Relationships between overflow indicators (velocity, temperature) and two potential predictors (AW inflow strength, sea level anomaly) show several strong correlations, although the underlying mechanisms remain to be fully resolved.

The manuscript clearly guides the reader through both similarities and differences across the investigated sites with well-structured descriptions of the background, methods, analyses, figures, and discussion. The statistical treatment is solid with clear reporting of significance levels and the conclusions are consistent with the evidence presented. I appreciate both the careful analysis of this massive dataset, the added value of the snapshot CTD sections and also the supplemental information. The interpretations are sensible, and the broader context is well articulated.

Overall, this study represents excellent progress in deciphering the flow structure across the ridge, reducing several long-standing uncertainties for this overflow region. I look forward to future monitoring efforts in this area, which will hopefully allow overflow transport estimates and further insights into the controlling mechanisms.

I have no major issues or questions but include a few comments and suggestions of various scope, intended to further improve the manuscript. I recommend acceptance after minor revision.

Thank you for your positive review. Below we respond to your individual comments and suggestions.

- - - Comments/suggestions for the text, by line number:

L72: Consider rephrasing from "…and no week during these two years had an average core velocity less than 30 cm s-1" to e.g. "…and throughout the entire record the weekly average core velocity remained above 30 cm s-1".
Thank you. The sentence has been rephrased following your suggestion.

L90: 'Should 'had' rather be 'has', and 'remaining' be 'remain' or 'remained'?
This sentence has been rephrased in response to R1.

L91: Simplify the "is joining in a…" to "According to Perkins et al.6, this water joins a focused flow over the northwestern part of the ridge and is transported towards the exit of the Western Valley (…).", which I think is what you mean.
This has been rephrased. Hopefully, it is clearer now.

L108: CTD not defined/spelled out earlier. Could write hydrographic observations here if it fits better with a definition on L150?
Thank you for noting this. We have followed your suggestion.

L127: Nice approach to use PVDs normalized by deployment duration although I'm confused about the resulting velocities being called 'residual velocities'? In a sense correct since they are based on de-tided velocities but confusing the context of PVDs. Aren't these velocities 'record-mean velocities', to be compared to/guided by the velocity scale shown? I must confess I have no good suggestion of a perfect term to use instead but please give this a thought and possibly adjust terminology.
Thank you for your comment. We agree that "residual velocities" probably are not a good term. We have decided to follow your suggestion and now use "record-mean velocities".

L165: "Overflow persistence" For me, persistence would also include the duration of an overflow event (e.g. the minimum number of consecutive days of such events?). At least for sites with quite normal distribution between inflow and outflow. (For the ones with high %, I guess it's already sign enough for a persistent overflow. Have you looked into that?
Thank you for your comment. We have replaced "Overflow persistence" with "Overflow stability", but with the same definition. We have also calculated Overflow stability for each of the sites and these results are presented in a new Table 1.

L185: "…bottom temperature was at or below 2oC at least half of the time, indicating overflow water." I take it you mean temperature at or below 2oC indicates overflow water, and not the "half of the time"? It would be good to support the 2oC with a reference.
The sentence was related to the histogram of bottom temperatures. That figure has been replaced with a new Figure 5 and the text modified. For the "2°C indicating overflow", we refer to TS-diagram in new Figure 2b.

L248: In the caption call (a)-(i) panels and not figure.
This has been done. Due to changes in other figures, this figure is now Figure 7.

L256: What is meant with '…almost complete coverage'. Is it the observations, or rather the number/network of instruments that could have been more complete?
It was supposed to mean "spatial coverage". The sentence has now been rephrased.

L258: "With that caveat, the observations provide a consistent picture." Do you mean Despite that caveat?
Yes. This has been corrected.

L369: Had to read twice (and check SupplTable1): clarify by rephrasing to e.g. "All ADCPs sampled at 20 min intervals, with one ping per ensemble for the BB ADCP and 12 pings per ensemble for the LR ADCP."
Thank you for your comment. We have used your suggestion with some slight modifications.

L373: I find a reference to the tidal analysis is lacking (is the analysis made by in-house scripts/routines?). Also, to guide the reader I would start with "To de-tide the velocities, a harmonic analysis is…"
We use in-house routines based on the Foreman FORTRAN package. This has been added to the text and a reference to Foreman (1978) has been added.

L379: How often did the SBE37/16 sample temperature?
At least every 20 minutes. This information has now been added.

L392: If not in the text (cf. L108, L150), spell out CTD here.
CTD is now spelled out in the text.

L393: SeaBird without dash
At L379 we use SeaBird and at L393 Sea-Bird. According to Sea-Bird's webpage (https://www.seabird.com/about), we believe this should be with a dash. L379 has therefore been corrected accordingly.

L393-394: Perhaps use 'were' instead of 'are'?
This has been corrected.

L399: Consider rephrasing from "For each of the ADCP sites, the closest grid point was found and daily SLA-values selected." to 'For each of the ADCP sites, daily SLA-values were extracted from the closest grid point. The distance between…'
The sentence has been rephrased following your suggestion.

L402: How was this extent determined? Just to fit the region properly, or by any other restrictions/features?
As noted in our response to R1, the original text was badly phrased. It has now been modified to emphasize that the link between sea level and overflow is mainly on the mesoscale. The region chosen for the "correction" was rather ad hoc. We just wanted a region surrounding the IFR on all sides and sufficiently large to represent the large-scale variations of sea level in the area.

L414: Just for clarity, one could add the scripting language/version used for the analysis?
Scripting language has been added.

- - - Comments/suggestions on the figures:

Figure 2: The PVDs, that are the most important and interesting features in the plot, don't stand out sufficiently and are easily confused with labels and the 500m-isobaths, at least when printed on paper (in digital format it is better since easier to magnify.) I wonder if black PVDs would be easier to follow, after all? And the bin-depth labels can be put in white textboxes. (One part of me would prefer seeing this on grey scale topography, as for Figure 1, which I think would make the important features stand out better.)Also, although you justify it in the captions, I find it hard when scales do not match between maps one is supposed to compare. Could both maps be drawn on the same scale after all?
Figure 2, which is Figure 3 in the revised version, has been redrawn so that the bathymetry is in grey scale shading, as suggested, and the PVD traces are orange. We have also tried to redraw panel b so that it has the same scale as panel a (which is the more important part of the figure). We were not, however, able to do that without the traces from different sites overlapping so much that the figure became confusing.

Figures 3, 4, and 5: Skip the panel labels (a, b, c, …). These are redundant and confusing in relation to the Site Letters, which are what you generally refer to in the text (and very well so). (Increase font size of the Site letter somewhat.)
Following Nature Communications guidelines (https://mts-ncomms.nature.com/cgi-bin/main.plex?form_type=display_auth_instructions), panels should be labelled using bold letters. But, we agree, that they are confusing in relation to the Site letters. We have therefore modified the figures in order to minimize the confusion.

Figure 6: Make panel (c) for the same temperature range as panel (a), for easier comparison?
The figure has been modified following your suggestion, also for panels (b) and (d).

Figure 7: nice 'snapshot' of a typical overflow!
Thank you!

Figure 8 (and Suppl.Figure 7): I was about to ask why the vertical and horizontal scales had to differ for the two transects (as stated in the caption). But it looks as if all panels indeed are on the vertical and horizontal same scale but not over the same ranges over depth and distance (since at different locations). Is that what you mean? Also, it would be helpful with indications of the CTD station locations, as in Figure 7 and Suppl.Figure 4.
Thank you for noting this. The panels do indeed have the same scales, but we had mistakenly kept the text in the caption referring to an earlier version of the figure. This has now been corrected. We have also added ticks, indicating locations of CTD stations to the figure (now Figure 7).

Suppl.Figure 4: Why different horizontal and vertical scales? It would be helpful to keep constant scales (although the ranges may vary for each site, cf. comment for Figure 8).
See reply above.

Suppl.Figure 6: In the caption, I think 'preferred' is a better word than 'selected': "…site E where no specific overflow direction seems to be selected." (BTW: Intriguing with such different results for relatively close site locations (and deployment periods!)
Corrected.

RESPONSE TO REVIEWERS' COMMENTS

We thank Reviewer #2 and #3 for taking their time to review the manuscript once more. We are glad that you very happy with the revision of the manuscript and how we responded to the comments from all three reviewers. A huge thank you to all three reviewers for providing constructive comments in the first round. The manuscript has now been modified following the editorial requests as well as the additional remarks from the reviewers. The changes are, however, not highlighted in the new version, since this was not a specific request from the Editor. Below we respond in red to the individual reviewer comments.

Reviewer #2 (Remarks to the Author):

Iceland-Faroe overflow revealed by in-situ observations
by Karin Margretha H. Larsen, Gudrid Eriksdottir, Bogi Hansen, Hjalmar Hatun, Steffen M. Olsen, and Svein Osterhus

The revised manuscript has been much improved by both the additions and removals of figures and text. In particular, the scatter plots in Fig. 5 are a nice addition. I have only a few minor comments below and hope to see this paper published very soon!

Thank you for reviewing the manuscript and once again thank you for the constructive comments in the previous round.

l.90 This sentence doesn't quite make sense as written and could be fixed in a few different ways. One possibility is to (a) substitute "combined" for "joint" and (b) add "is" after "water".
Thank you for the comment. The sentence has been updated accordingly.

l.241 Change "Fig. 6" to "Fig. 7"
Corrected.

l.260-261 "transport per unit width" is a clearer label than "transport density". I'd suggest sticking with the former.
We agree. The text has been modified following your suggestion.

l.288-291 Since the Eastern and Western pathways seem to be somewhat anti-correlated, it's likely that the summed transport of the two is a more stable value which could be stated with greater confidence.
We have added a sentence to address this (see new line 230-232).

l.324-325 Isn't it more common to state the $p<0.05$ result as "different from zero with 95% confidence", rather than "at the 5% level"? Same for 99% and 99.9%. That would make more sense to me (though I suspect readers can figure it out).
We agree. The text has been modified following your suggestion.

Reviewer #3 (Remarks to the Author):

Dear Authors, and Editors,

Thank you to the Authours for the thorough revision of your manuscript and clear response back to all reviewers. I find you have solidly implemented the comments by us all (or, in a few cases, provided well motivated justifications when keeping things 'as is'), and arrived at more solid and well-balanced presentation of your valuable study.

I particularly value the re-worked section "Controlling mechanisms", the move of the original Fig7 to now Fig2, the inclusion of Table 1 with ADCP characteristics plus estimates, and the new Fig5 presenting combined numberd from the original Figures 4 & 5 (and I hope the original histograms in fig5 can be revisited in upcoming analyses?).

I also appreciate the work on arriving at the transport estimates (or at least as-close-to-transport-estimates-as-possible) for the main branches, as requested by R1 and R2. I think the authors do a good job motivating their approach and choices for this analysis, and also being clear about the related (high) uncertainties. Hopefully, future monitoring will provide more secure estimates.

The additions and revisions to the text, sections/headers related to the above modifications are satisfactory, addressing the comments from all reviewers. Also smaller changes, such as the redistribution of the panels/sites in the current Figs4 and 5, has improved the presentation.

For my part, I recommend the manuscript to be accepted for publication, subject to only a few technical corrections (cf. the below comments).

Thank you for taking time to review the manuscript again and thank you for providing additional corrections and suggestions for improvements.

I noted down a few things that could be worth a check, for improved readability,

Manuscript:

L61 "The most perplexing unresolved question, however, ..." ?
Corrected.

(L70 mistyping around the Beaird9 ref, L74+L75 reformat cm s-1)
Corrected.

L135 A bit convoluted. Sufficient with: "Here, we use daily averaged velocities measured by the ADCPs at several levels ("bins") above the instrument."?
Corrected.

L144 In fig3 caption, simplify to: "The orange traces show PVDs based on daily averaged velocities for (a) the deepest bin, Bin-1, and (b) the Top-bin (see text and Methods). At the end of each PVD is shown the centre depth for (a) Bin-1 and (b) the Top-bin. "
Corrected.

L170 "Main characteristics for the seven sites are listed in Table 1, which illustrates the dominance

of the three main overflow sites: ...". Not entirely evident (from the table readings) which properties you define the dominance by (e.g. "with regard to average velocity...")
You are correct. The sentence now reads “Main characteristics for the seven sites are listed in Table 1, where especially the average overflow velocity illustrates the dominance of the three main overflow sites”. Hopefully this version is clearer.

L328 "The bottom four rows..." or "The last four rows..."?
Corrected.

The Supplement:
Supplementary Table 1. Since the ADCPs are all moored deep it is perhaps evident, but could be good to clarify that all ADCPs were "upward looking". This would also aid the bin-explanation in L135-138. May also be invoked in L400: "The upward looking ADCPs were either placed...".
“Upward looking” has been added at L400, L135 as well as the Supplementary Table 1 Caption.

Supplementary Fig. 5. Is it possible to include the average profiles form Fig.4 here (e.g. as thick white line?), as aid to the reader since both the x- and y-ranges have changed?
The average profiles have been added to the figure.
